# Subchondral bone influences chondrogenic differentiation and collagen production of human bone marrow-derived mesenchymal stem cells and articular chondrocytes

**DOI:** 10.1186/s13075-014-0453-9

**Published:** 2014-10-07

**Authors:** Michaela Leyh, Andreas Seitz, Lutz Dürselen, Jens Schaumburger, Anita Ignatius, Joachim Grifka, Susanne Grässel

**Affiliations:** Department of Orthopedic Surgery, University of Regensburg, ZMB/BioPark 1, Josef-Engert-Str. 9, Regensburg, 93053 Germany; Centre for Medical Biotechnology, BioPark 1, Josef-Engert-Str. 9, Regensburg, 93053 Germany; Institute of Orthopedic Research and Biomechanics, Centre of Musculoskeletal Research, University of Ulm, Helmholtzstr. 14, Ulm, 89081 Germany

## Abstract

**Introduction:**

Osteoarthritis (OA) is characterized by an imbalance in cartilage and underlying subchondral bone homeostasis. We hypothesized that signals from the subchondral bone may modulate production of matrix components, alter chondrogenic differentiation potential of cocultured bone marrow-derived mesenchymal stem cells (BMSC) and induce a phenotypic shift in differentiated OA chondrocytes.

**Methods:**

We established a novel coculture model between BMSC, mixed cultures (BMSC and chondrocytes) and chondrocytes embedded in fibrin gel with OA and normal subchondral bone explants (OAB and NB). Tissues and cells were either derived from OA or trauma patients. In addition, we used adipose-derived stem cells (ASC) from liposuction. With gene expression analysis, biochemical assays, immunofluorescence and biomechanical tests we characterized the properties of newly generated extracellular matrix (ECM) from chondrocytes and chondrogenically differentiating BMSC cocultured with OAB or NB in comparison with monocultures (cultures without bone explants).

**Results:**

Overall, gene expression of collagens of OAB and NB cocultured cells was reduced compared to monocultures. Concomitantly, we observed significantly lower collagen I, II and III and glycosaminoglycan (GAG) production in OAB cocultured cell lysates. In parallel, we detected increased concentrations of soluble GAGs and basic fibroblast growth factor (bFGF), interleukin (IL)-6 and IL-8 in supernatants of OAB and NB cocultures mainly at early time points. IL-1ß concentration was increased in supernatants of OAB cocultures, but not in NB cocultures. Cell-free NB or OAB explants released different amounts of IL-1ß, bFGF and soluble GAG into cell culture supernatants. In comparison to cocultures, monocultures exhibited higher Young’s modulus and equilibrium modulus. Stimulation of monocultures with IL-1ß led to a downregulation of aggrecan (*ACAN*) gene expression and in general to induced matrix metalloprotease (*MMP*)*2, MMP3* and *MMP-13* gene expression while IL-6 and IL-8 stimulation partly reduced *ACAN*, *MMP3* and *MMP-13* gene expression.

**Conclusions:**

Our results suggest an alteration of molecular composition and mechanical properties of the newly formed ECM in subchondral bone cocultures. We suggest that soluble factors, that is interleukins and bFGF, released in cocultures exert inhibitory effects on collagen and temporary effects on proteoglycan production, which finally results in a reduction of mechanical strength of newly formed fibrillar networks.

**Electronic supplementary material:**

The online version of this article (doi:10.1186/s13075-014-0453-9) contains supplementary material, which is available to authorized users.

## Introduction

For long-term repair and regeneration of focal cartilage defects, chondrocytes are implanted at the site of injury, however, not much attention has been paid to the microenvironmental effects of neighboring cartilage/subchondral bone. This is specifically evident in diseases affecting diarthrodial joints such as osteoarthritis (OA), which is an age-related and/or trauma-induced multifactorial, slowly progressing and primarily noninflammatory degenerative disorder of the synovial joints culminating in the irreversible destruction of the articular cartilage [1,2].

Research has focused on chondrocytes and cartilage as mediators of OA but also other cells and tissues of the joint-like synovium or subchondral bone are known to be involved in OA-pathogenesis. There is strong evidence for bone changes during OA progression: increased turnover of subchondral bone, thinning trabecular structures, sclerosis of the subchondral plate, bone marrow lesions and subchondral bone cysts [3,4]. Other studies showed alterations in the collagen turnover and cytokine release of osteoarthritic subchondral bone matrix [5,6].

Therapies using adult bone marrow-derived mesenchymal stem cells (BMSC) have a promising future to facilitate regenerative musculoskeletal tissue repair. Especially, BMSC are identified as a relevant cell source for regeneration of focal cartilage and bone lesions, because they can be readily expanded *in vitro* - whereas differentiated cells, that is chondrocytes dedifferentiate upon expansion [7]. BMSC are pluripotent cells that inherit the capacity to differentiate into cartilage, bone, fat, and other tissue types after appropriate *in vitro* induction [8].

So far, OA-related cartilage lesions and fissures have not been a widely clinically approved target for BMSC-based therapies as this would imply to implant cells into the neighborhood of diseased tissue where they are confronted with an altered microenvironment of the neighboring pathological cartilage and subchondral bone tissue. It has been demonstrated that BMSC are able to differentiate into a specific cell phenotype depending on the environment they are actually residing in. Crosstalk between BMSC and extracellular matrix (ECM) components could be a crucial determining factor for the differentiation of BMSC into chondrocytes [9]. Indeed, the microenvironment of OA subchondral bone (OAB) is likely to have an influence on the ability of BMSC to regenerate articular cartilage or subchondral bone matrix as implanted stem cells may respond in a different way to differentiation stimuli due to signaling factors secreted from neighboring OA chondrocytes or osteoblasts [10].

One way to direct and redirect the differentiation of BMSC are coculture systems that promote diffusion of secreted paracrine factors and cell-cell interactions [11,12]. Westacott *et al.* demonstrated that subchondral osteoblasts are able to modulate the metabolism of chondrocytes and change their phenotype [13]. Of note, the ratio of cocultured BMSC and articular chondrocytes regulate whether differentiation proceeds toward a cartilaginous or osseous phenotype. Culturing articular chondrocytes with BMSC in a 2:1 ratio induces both phenotypes simultaneous in a three-dimensional alginate hydrogel construct indicating that chondrocytes provide the necessary factor(s) in this process [14]. These osteoinductive properties of articular chondrocytes were also reported for a culture model where chondrocytes in alginate hydrogels were cocultured above a monolayer of BMSC preventing direct cell-to-cell contact. Notably, the effect was time-dependent, the longer the coculture period the more prolonged and stable was the osteogenic phenotype of BMSC [15].

However, the underlying mechanisms of cell-cell interactions in OA joint tissues that influence chondro-osteogenic differentiation of BMSC have not been elucidated nor fully understood. Also, the identity of factors from OAB bone tissue and cells that might modulate the chondrogenic phenotype of BMSC is not comprehensively delineated. We address this subject in the present study where we have established a novel *in vitro* coculture model to evaluate the influence of subchondral bone from OA-affected joints on chondrogenic differentiation of human BMSC derived from OA patients, the phenotype of differentiated OA chondrocytes and the properties of newly synthesized ECM. In addition, we have included a ‘triculture’ model consisting of a mixture of chondrocytes and BMSC cultured on subchondral bone explants. In order to attribute effects to disease status and cell source, we reproduced key experiments using a coculture regimen with adipose-derived stem cells and ‘normal’ subchondral bone explants from trauma patients. Here, we aim to test if cell-to-cell contact between differentiated chondrocytes and undifferentiated BMSC compensate effects of factors from subchondral bone on chondrogenic differentiation of BMSC. To provide a chondrogenic favorable environment, cells were embedded in fibrin gel [16], seeded onto the surface of subchondral bone explants and kept in chondrogenic medium for up to 28 days.

## Material and methods

### Culture and isolation of human subchondral bone explants, BMSC and chondrocytes

Human articular cartilage was collected from surgically removed joints of patients undergoing total knee replacements (TEP) due to OA. This had been approved by the local ethics committee (Az: 08/065; Ethikkommission an der Universität Regensburg, email: ethikkommission@klinik.ukr.de) and specimens were taken with patients’ written consent. For this study, knee joints were obtained from 32 different donors (13 male and 19 female, mean age 67 ± 9). Prior to culture, the cartilage surface of surgically removed tissue was classified macroscopically as either damaged or intact according to a predefined procedure comprising color, surface integrity and tactile impression tested with a standard scalpel [17]*.* We used only healthy-appearing pieces for isolation of chondrocytes and generation of subchondral bone chips. OAB chips were produced as follows: surgically removed tissue was thoroughly washed with phosphate-buffered saline (PBS). Then the cartilage surface was scored as described above and only justly evaluated pieces were accepted for usage. Cartilage was cut off for isolation of chondrocytes and the completely denuded bone was used for generation of explants of 8 mm in diameter and 4 mm height. Chondrocytes were isolated from cartilage slices upon overnight digestion with collagenase II (PAA, Piscataway, NJ, USA) at 37°C. Isolated cells were resuspended in Dulbecco’s modified Eagle’s medium (DMEM; Gibco Invitrogen, Paisley, UK) containing 10% fetal calf serum (FCS; Sigma-Aldrich, St. Louis, MO, USA) and 1% penicillin/streptomycin (PAA, Piscataway, NJ, USA). Chondrocytes were kept in monolayer in an initial density of 17,000 cells per cm^2^ cultured in a humidified 37°C/5% CO_2_ incubator for 7 to 14 days and were used when confluent (passage 1). Normal subchondral bone tissue (NB) was received from knee joints of rare trauma-affected patients (treated for sport accidents, two male and two female, mean age 35 ± 11) and were prepared as described above. The bone tissue used in this study was not injured directly but was collected during the surgical treatment of the patient from another nonaffected region of the same joint. Preparation of bone explants for culture was similar for OA- and trauma-derived bone tissue.

All human BMSC used for this study have been isolated from bone marrow aspirates obtained from a total number of 30 different patients (14 male and 16 female, mean age 61 ± 8) undergoing a hip replacement surgery due to OA. This had been approved by the local ethics committee (Az: 08/065; Ethikkommission an der Universität Regensburg, email: ethikkommission@klinik.ukr.de) and specimens were taken with patients’ written consent. The bone marrow was centrifuged and cells were fractionated on a density gradient (Biocoll separating solution; Biochrom, Berlin, Germany). The low-density cell fraction concentrated in the interphase (‘buffy coat’) was washed, seeded in cell culture flasks supplied with MesenchymStem Medium (PAA, Piscataway, NJ, USA) and nonadherent cells were removed after 5 to 7 days. Adherent cells were cultured until they reached approximately 80% confluence. After splitting, BMSC were seeded at a density of 4 × 10^4^ cells/cm^2^ and kept in culture for up to three passages before they were used for experiments. At this passage, BMSC were positive for CD44 and CD105 and negative for CD19 and CD34. Additionally, we analyzed their differentiation capacity into osteogenic and adipogenic lineages. A representative specimen is shown in Figure S2 in Additional file 1.

Adipose-derived stem cells (ASC) (isolated as described previously by [18]) were isolated from subcutaneous fat tissue that was obtained from patients undergoing elective body-contouring procedures. Five different patients were used for our coculture setup (female, age <50). Written consent to harvest ASC from patients was obtained from the local ethics committee (Az: 08/117; Ethikkommission an der Universität Regensburg, email: ethikkommission@klinik.ukr.de).

Briefly, fat tissue was washed, minced and digested in serum-free MEM (1 ml/1 g tissue) with Liberase Blendzyme 3 (2U/1 g tissue; Roche Diagnostics, Basel, Switzerland) at 37°C for 45 min. The lysate was filtered (100- and 40-μm filters; Fisher Scientific, Schwerte, Germany) and centrifuged at 450 x g for 10 min. The cell pellet was washed twice with Hanks’ balanced salt solution (Cellgro, Manassas, VA, USA) and cells were seeded in culture vials (Greiner Bio-one, Frickenhausen, Germany) and daily washed to remove unwanted red blood cells or nonadherent cells. After reaching a confluence of 80%, ASC were seeded at a density of 3,000 cells/cm^2^, maintained in ASC medium (αMEM containing 20% FCS, 2 mM L-glutamine and 1% penicillin/streptomycin, Sigma-Aldrich, St. Louis, MO, USA) and used for experiments at passages 5.

ASC were characterized in accordance with guidelines of the Declaration of Helsinki for biomedical research from the Applied Stem Cell Research Center of the University of Regensburg.

**Figure 1:**
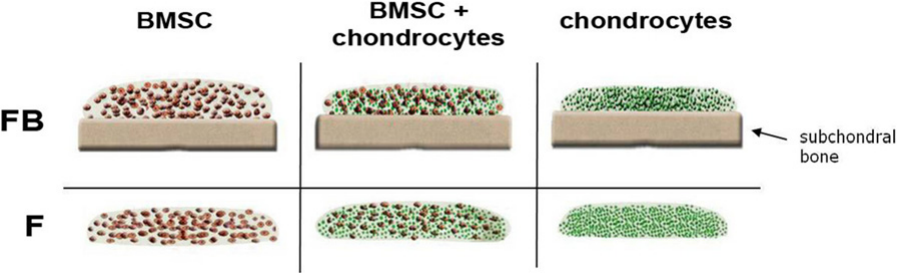
**Model of contact coculture between cells and subchondral bone explants.** BMSC, mixed cultures (BMSC + chondrocytes 1:1) and chondrocytes were embedded in fibrin gel and these cell-gel constructs were applied onto the surface of subchondral bone explants (co- and tricultures). As controls, cells were embedded in fibrin gels and cultured without subchondral bone (monocultures). All experimental setups were kept for up to 28 days in chondrogenic medium. Samples were harvested at days 7 and 28. BMSC, bone marrow-derived mesenchymal stem cells; F, fibrin gel-embedded monocultures (without subchondral bone explants); FB, fibrin gel-embedded co- and tricultures together with subchondral bone explants.

A suspension of fibrinogen (10 μl, 100 mg/ml, Sigma-Aldrich, St. Louis, MO, USA) and 1 × 10^6^ BMSC, 1 × 10^6^ ASC, 2 × 10^6^ chondrocytes or a mixture of 5 × 10^5^ BMSC and 5 × 10^5^ chondrocytes (1:1) was homogenously mixed with thrombin (18 μl, 5 U/ml; Baxter, Munich, Germany). Because of the smaller size of chondrocytes, their cell number had to be adjusted to obtain a comparable volume of fibrin gel and to avoid effects due to different medium diffusion capacity resulting in an altered maintenance with nutrients. The cell-fibrinogen suspension was applied onto the surface of either OAB or NB explants (co- and tricultures) or as a droplet on the bottom of a 24-well plate (monocultures) (Figure 1). Full polymerization was reached after 45 min at 37°C and resulted in a stable and clear hydrogel with a pore size of approximately 50 μm. Cell-free OAB and NB explants were included into our setups. Mono-, co- and tricultures as well as cell-free bone explants were kept in chondrogenic medium in the presence of transforming growth factor ß (TGF-ß)-3 (10 ng/ml, R&D Systems, Minneapolis, MN, USA) and cultured for up to 4 weeks [19]. After 7 and 28 days, a specimen of co- or tricultured fibrin gels was carefully separated from subchondral bone explants using a thin spatula. Fibrin gels were processed for histology, immunofluorescence, biochemistry, biomechanical or gene expression analysis as described below. Culture supernatants of day 7 and 28 were collected and frozen at -80°C until usage.

### Cell vitality assay in fibrin gel setups

In order to evaluate vitality of cells in our different culture setups, lactate dehydrogenase (LDH) concentration in supernatants of BMSC, mixed cultures and chondrocytes was determined in monocultures and co- or tricultures with OAB kept in chondrogenic medium. Content of LDH was analyzed at days 7, 14, 21 and 28 with an LDH-based cytotoxicity detection kit (Roche Diagnostics, Penzberg, Germany) and compared to respective assay controls (high control = all cells in fibrin gels were lysed; low control = spontaneous cell death of an equivalent cell amount in monolayer) according to the manufacturer’s instructions. LDH concentration released from dead cells into supernatant was determined on a photometrical basis at absorption of 490 nm (Tecan GENios with Magellan 6.5; Tecan, Crailsheim, Germany). Due to high interexperimental variability - presumably caused by personal living conditions and physical activity, medical treatment or general health status of tissue donors - we have calculated the raw data as percentage of control per individual experiment. Repetition was in triplicate at least three times with cells and explants from different donors.

### Histology and immunofluorescence of fibrin gel-embedded cultures

Fibrin gels were rinsed with PBS (PAA, Piscataway, NJ, USA), embedded in TissueTec (Sakura Finetek, Alphen aan den Rijn, the Netherlands) and were frozen in liquid nitrogen. For histological evaluation, sections (10-μm thick) were fixed in 4% paraformaldehyde (PFA: Sigma-Aldrich, St. Louis, MO, USA) for 10 min at RT, stained with 1% Alcian blue 8GX (Sigma-Aldrich, St. Louis, MO, USA) for glycosaminoglycans (GAGs) and counterstained with nuclear fast red aluminium sulfate solution (Roth, Arlesheim, Germany). For immunofluorescence analysis, sections were fixed in 4% PFA for 10 min at RT, treated for 30 min at 37°C with pepsin (3 mg/ml in 0.01 M HCl, Sigma-Aldrich, St. Louis, MO, USA) and were blocked with 3% bovine serum albumin (BSA) (Sigma-Aldrich, St. Louis, MO, USA) diluted in PBS for 1 h. Sections were labeled overnight at 4°C with an antibody against collagen I (C-2456, Sigma-Aldrich, St. Louis, MO, USA), collagen II (CIIC1; DSHB, Iowa City, IA, USA) or collagen III (MAB 3392; Merck Millipore, Billerica, MA, USA). In case of collagen X staining (2031501005; Quartett, Berlin, Germany) a hyaluronidase (Sigma-Aldrich, St. Louis. MO, USA) pretreatment instead of pepsin was used. For fluorescence detection, all sections were incubated with an Alexa Fluor™ 488 secondary antibody (Invitrogen, Paisley, UK) for 1 h at 37°C. Finally, samples were mounted in Vectashield mounting medium with DAPI (1 μg/ml, Vector Laboratories, Burlinton, ON, Canada) and were analyzed with an Olympus BX 61 imaging system and cell^P^ software (Olympus, Hamburg, Germany). Repetition was at least five times with cells and explants from different donors.

### Biochemical analysis of fibrin gel cell lysates and supernatants

Fibrin gels were homogenized and digested with pepsin (1 mg/ml in 0.5 M acetic acid containing 0.4 M NaCl, Sigma-Aldrich, St. Louis, MO, USA) for 48 h at 4°C and further digested with elastase (1 mg/ml in Tris-buffered saline (TBS) pH 8; Serva Electrophoresis, Heidelberg, Germany) for 24 h at 4°C. Samples were stored at -20°C until they were analyzed for GAGs and collagen contents.

GAG concentration was measured spectrophotometrically using 25 μl of the digested cell lysates or 25 μl undiluted cell supernatant supplemented with dimethylmethylene blue (DMMB; AppliChem, Darmstadt, Germany), which forms a complex with GAGs. Quantification was performed in μg per 1 × 10^6^ cells with a chondroitin sulfate standard at 525 nm (Tecan GENios with Maggelan 6.5; Tecan, Crailsheim, Germany).

Collagen I and II contents of day 28 cell lysates digested as described above were measured with specific sandwich enzyme-linked immunosorbent assays (ELISAs), which recognize the native conformation of collagen I and II chains (Chondrex, Redmond, WA, USA), according to the manufacturer’s instructions.

Collagen III in digested cell lysates was quantified with a dot blot assay using 1 μl from 500 μl of cell lysate and including a standard curve of recombinant collagen III (Abcam, Cambridge, UK). Quantification was performed densitometrically in μg per 1 × 10^6^ cells using a Chemi-Smart 500 (PeqLab, Erlangen, Germany) for fluorescence detection and the CS4 Windows software for calculation of luminescence intensities. Repetition was in triplicate at least five times with cells and explants from different donors.

### RNA isolation and real-time PCR amplification

Cell-fibrin gel suspensions suspended in peqGOLD TriFast (PeqLab, Erlangen, Germany) were minced and RNA was isolated according to a Trizol protocol, followed by column purification with the absolutely RNA Microprep Kit (Agilent Technologies Stratagene, Santa Clara, CA, USA) according to the manufacturer’s instructions. cDNA was generated from 500 ng of RNA using AffinityScript QPCR cDNA Synthesis Kit and oligo(dT) primers (Agilent Technologies Stratagene, Santa Clara, CA, USA) according to the manufacturer’s instructions. Repetition was in triplicate at least five times for BMSC, mixed and chondrocytes cocultures with bone explants from different OA donors and at least four times for ASC cultures.

Quantitative real-time PCR was performed in triplicate using 30 ng cDNA (RNA equivalent) and qPCR master mix SYBR Green Dye I on MxPro-Mx305P (Agilent Technologies Stratagene, Santa Clara, CA, USA). For quantification, a plasmid standard curve for *COL1A1, COL2A1, COL3A1* and *COL10A1* was included on each PCR plate (plasmid copy ranges for *COL1A1:* 1 × 10^6^ to 1 × 10^2^; *COL2A1:* 3.4 × 10^7^ to 3.4 × 10^3^; *COL3A*1: 1 × 10^6^ to 1 × 10^2^ and *COL10A1:* 5 × 10^4^ to 5 × 10^0^). Gene expression of matrix metalloproteinase (*MMP*)*2, MMP3, MMP13* and aggrecan (*ACAN*) gene was normalized with *GAPDH* and data evaluation was relative employing the ΔΔCT method using gene expression of unstimulated monocultures as calibrator. Data analysis was carried out with the MxPro QPCR 4.0 software (Agilent Technologies Stratagene, Santa Clara, CA, USA). The following forward and reverse primer pairs were used for gene expression analysis: for *COL1A1* 5′-AGC TCC TGG TGA AGT TGG TC-3′ and 5′-ACC AGG GAA GCC TCT CTC TC-3′; for *COL2A1* 5′-TGC TGC CCA GAT GGC TGG AAG A-3′ and 5′-TGC CTT GAA ATC CTT GAG GCC C-3′; for *COL3A1* 5′-GTC CAT GGA TGG TGG TTT TC -3′ and 5′-GTG TGT TTC GTG CAA CCA TC-3′; for *COL10A1* 5′-CCC TCT TGT TAG TGC CAA CC-3′ and 5′-AGA TTC CAG TCC TTG GGT CA-3′; for *MMP2* 5′-GCC AAT GGA GAC TGT CTC AAG A-3′ and 5′-TTC TAA GGC AGC CAG CAG TGA A-3′; for *MMP3* 5′-AAC CTG TCC CTC CAG AAC CT-3′ and 5′-GGAAGAGATGGCCAAAATGA-3′; for *MMP13* 5′-CAC CGG CAA AAG CCA CTT-3′and 5′-TAGAC TGG TAA TGG CAT CAA GGG A-3′; for *ACAN* 5′-CTA TAC CCC AGT GGG CAC AT-3′ and 5′-GGC ACT TCA GTT GCA GAA GG-3′and for *GAPDH* 5′-ACC CAG AAG ACT GTG GAT GG-3′ and 5′-TTC TAG ACG GCA GGT CAG GT-3′.

### Analysis of soluble collagens in culture supernatants with hydroxyproline assay

The amount of total soluble collagen in culture supernatants was determined by the Total Collagen Hydroxyproline Assay (QuickZyme Biosciences, Leiden, Netherlands) according to the manufacturer’s protocol. Briefly, after 3 days, 1 ml culture supernatant from monocultures or subchondral bone cocultures was harvested and soluble collagens in the supernatant were hydrolyzed into amino acids (12 M HCl for 20 h at 95°C). Hydroxyproline was stained and color formation was quantified at absorbance maximum at 570 nm (Tecan GENios with Maggelan 6.5; Tecan, Crailsheim, Germany). Repetition was in triplicate at least five times with cells and explants from different donors.

### Analysis of culture supernatants for bFGF, IL-1ß, IL-6 and IL-8

To determine the concentration of specific proteins in the supernatant, human interleukin-1ß (IL-1ß) sandwich ELISA kit (Ray Biotech, Inc., Norcross, GA, USA), interleukin-6 (IL-6) sandwich ELISA kit (R&D Systems, Minneapolis, MN, USA), interleukin-8 (IL-8) ELISA kit (Gen-Probe, Bedford, MA, USA) and basic fibroblast growth factor (bFGF) sandwich ELISA kit (R&D Systems, Minneapolis, MN, USA) were used according to the manufacturer’s instructions. Repetition was in triplicate at least six times for BMSC, mixed cultures and chondrocytes cocultured with bone explants from different OA donors and at least four times for cell-free OA and normal bone explants as well as for ASC cultures.

### Biomechanical testing

Samples with a standardized outer diameter of 2.6 mm were punched out of the fibrin gel-cell constructs (biopsy punch, Stiefel, Munich, Germany). The biomechanical tests were carried out in a standard material testing machine (Z010; Zwick, Ulm, Germany) equipped with a 40 N load cell. The initial sample height (h_0_) was assessed under a preload of 0.1 N by use of a laser displacement transducer (optoNCDT 2200-20; Micro-Epsilon Messtechnik GmbH & Co., Ortenburg, Germany, 0.3 μm resolution, ±0.03% accuracy). Subsequently, the samples were placed in a cell culture dish filled with 0.9% NaCl and an unconfined compression test was performed by loading it through a flat-ended punch at a strain rate of 100% h_0_/min until 50% strain was reached. The Young’s modulus was then calculated at two typical regions from the stress-strain diagrams (progressive region at 0 to 10% strain and the linear region at 40 to 50% strain). The samples were then stored for 24 h at 4°C in physiological saline to let them completely recover. After that, an additional stress-relaxation test was performed. The samples were placed in a confining chamber of 2.6 mm diameter filled with 0.9% NaCl and loaded by a porous ceramic cylinder (Al_2_O_3_) allowing for fluid exudation. Fifty percent strain was applied at a strain rate of 100% h_0_/min and held constant for 10 min until the equilibrium state was reached. Then the hydraulic permeability (k) was determined using a least squares algorithm derived from a diffusion equation [20,21] (Formula 1), where Δl/h_0_ is the applied strain and t the time. The aggregate modulus (H_A_) at equilibrium state at 50% strain was calculated as described in Formula 2 where σ_***∞***_ was the equilibrium stress at a strain of 50%. Repetition was four times with cells and explants from different donors.

$$ \begin{array}{c}\hfill \mathrm{Formula}\kern0.15em 1\kern0.24em {\sigma}_{\infty }+2\kern0.24em H\kern0.24em *\frac{\varDelta 1}{h_0}\kern0.24em *\kern0.24em {e}^{\left({\left(\frac{\pi }{h_0}\right)}^2\kern0.24em *\kern0.24em H\kern0.24em *K\kern0.24em *\kern0.24em t\right)}\hfill \\ {}\hfill \mathrm{Formula}\kern0.15em 2\kern1.12em {H}_A=\frac{\sigma_{\infty }}{\varepsilon_{50\%}}\hfill \end{array} $$

### Stimulation of BMSC, mixed cell populations and chondrocytes embedded in fibrin gel

Stimulation of monocultures with IL-1ß (5 ng/ml, Biomol, Hamburg, Germany), IL-6 (5 ng/ml, RayBiotech, Norcross, GA, USA) or IL-8 (10 ng/ml, RayBiotech, Norcross, GA USA) was performed in chondrogenic medium for the first 7 days. Fibrin gels were harvested after 7 or 28 days and processed for gene expression analysis as described above. Repetition was in triplicate at least five times with cells and bone explants from different donors.

### Culture setups of ASC with subchondral bone

ASC were seeded onto the surface of OAB or NB explants as described above. Fibrin gels of days 7 and 28 were processed for qPCR and culture supernatants were collected and frozen at -80°C until usage for analysis of cytokines, bFGF and GAG.

### Stimulation of BMSC with conditioned medium

BMSC monocultures were supplemented either with fresh chondrogenic medium or with chondrogenic medium that was conditioned for three days by OAB explants. Monocultures and stimulated cultures were kept under these conditions for up to 28 days and medium was changed every 3 days. After 7 and 28 days, fibrin gels were processed for gene expression analysis as described above. Repetition was in triplicate at least four times with cells and explants from different donors.

### Statistical analysis

All results are calculated for an initial cell number of 1 × 10^6^ cells. The mean standard deviation (SD) values were calculated for all variants. The nonparametric Wilcoxon test (for paired analyses) or the Mann-Whitney test (unpaired analyses) was applied to analyze differences between time points and between culture conditions. All experiments were performed in triplicate and repeated at least four times with cells from different donors. *P* <0.05 values were considered to indicate statistically significant differences. Data analysis and graphing was performed with GraphPad for Windows version 5 (GraphPad Software, Inc., La Jolla, CA, USA).

## Results

### Viability of cultured cells

In order to evaluate if the fibrin gel system affects vitality/viability of cells, LDH concentration in culture supernatants was analyzed and compared to respective monolayer controls. Chondrocytes, BMSC and mixed cultures embedded in fibrin gel cocultured or kept as monocultures showed no enhanced LDH release compared to assay controls. We therefore assume that cells are not influenced in their vitality or overall metabolic activity by fibrin gel components or subchondral bone explants during the culture time period (Figure S1A-C in Additional file 2).

### Culture models

We set up different culture conditions to analyze the influence of factors from subchondral bone on multipotent BMSC with respect to ECM formation and chondrogenic differentiation. Therefore, we cocultured BMSC embedded in fibrin gel with subchondral bone explants from OA patients. As a control for chondrogenic properties of BMSC we used cocultures of differentiated articular chondrocytes and mixed cultures (tricultures) of chondroctyes and BMSC (both embedded together in fibrin gels) to include putative cell-to-cell effects of differentiated cells on undifferentiated cells. As a control for cocultures we set up the respective monocultures of cells embedded in fibrin gels and cultured in chondrogenic medium (Figure 1). Additionally, we analyzed cell-free subchondral bone explants from OA and trauma donors with respect to cytokines, soluble (s)GAG and bFGF release and included an ASC coculture set up with OAB and NB explants with respect to gene expression and release of cytokines, sGAG and bFGF.

### Collagen gene expression of cocultures versus monocultures

To determine whether factors released from subchondral bone (OAB or NB) affect matrix production or chondrogenic differentiation of ASC or BMSC and chondrogenic phenotype of chondrocytes, mRNA expression of *COL1A1* (dedifferentiation marker)*, COL2A1* (marker for chondrogenic differentiation)*, COL3A1* (mesenchymal marker) and *COL10A1* (marker for chondrocyte hypertrophy) was analyzed at days 7 and 28.

**Figure 2:**
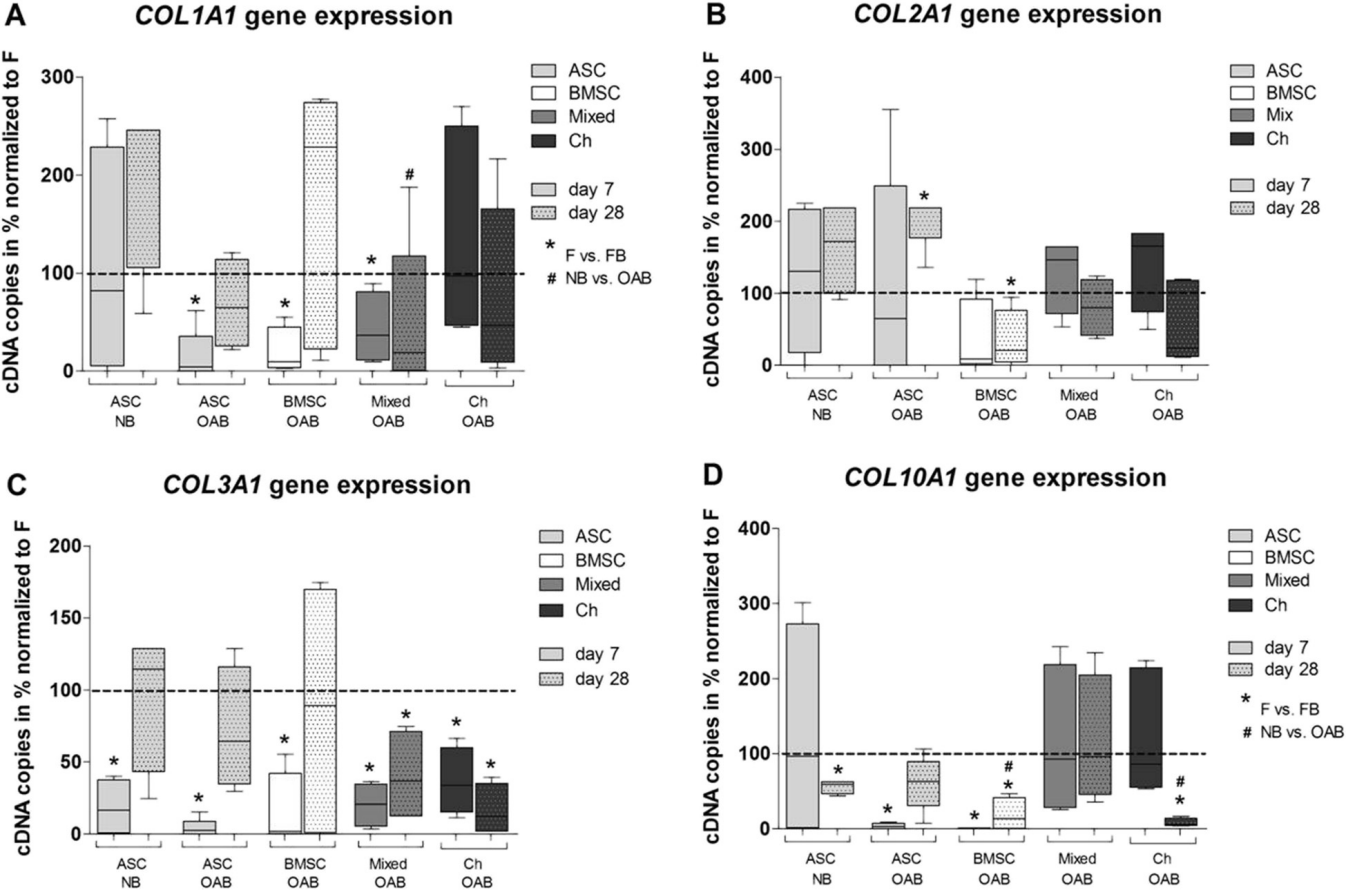
**Quantification of gene expression with qPCR.** Gene expression level of **(A)** *COL1A1,* **(B)** *COL2A1,* **(C)** *COL3A1* and **(D)** *COL10A1* were determined in mono-, co- and tricultures at day 7 (blank bars) and day 28 (bars with pattern) using plasmid standard curves. ASC (light grey bars), BMSC (white bars), mixed cultures (BMSC and chondrocytes in a ratio of 1:1, dark grey bars) and chondrocytes (black bars) were kept as monocultures (dotted lines at 100%) or as co- and tricultures (FB) with NB or OAB explants in chondrogenic medium. Due to high interexperimental variability we have calculated the raw data as a percentage of highest cDNA copy number per individual experiment and to 100% of monocultured controls. Stars (^*^) indicate significant differences between mono- and cocultures, hash signs (^#^) indicate significant differences between NB and OAB cocultures. Results are mean with standard deviation (SD). N =5; ^*^ *P* <0.05, ^**^ *P* <0.01, ^***^ *P* <0.001. ASC, adipose-derived stem cells; BMSC, bone marrow-derived mesenchymal stem cells; FB, fibrin gel-embedded co- and tricultures together with subchondral bone explants; NB, normal subchondral bone; OAB, osteoarthritic subchondral bone.

At day 7, we observed a significant inhibition of *COL1A1* gene expression in ASC and BMSC cocultured with OAB and in mixed tricultures compared to monocultures, whereas ASC cocultured with NB and chondrocyte OAB cocultures remained unaffected. At day 28 none of the culture conditions that affected *COL1A1* gene expression was statistically significant. ASC cocultured with NB had higher mRNA level of *COL1A1* compared to mixed tricultures at day 28 while all other OAB coculture conditions revealed no significant difference to NB cocultures (Figure 2A). Analyses of *COL2A1* gene expression in OAB cocultures revealed a significant upregulation in ASC cocultures and a significant downregulation in BMSC cocultures compared to respective monocultures at day 28. *COL2A1* gene expression in all other culture regimen remained unaltered. No significant differences in *COL2A1* gene expression were detectable between NB and OAB cocultures (Figure 2B).

In all co- and tricultures *COL3A1* gene expression was significantly downregulated at day 7 and in mixed tricultures and chondrocyte cocultures also at day 28 compared to monocultures. No significant differences in *COL3A1* gene expression were detectable between NB and OAB cocultures (Figure 2C).

*COL10A1* gene expression was reduced in NB cocultures with ASC at day 28 and in OAB cocultures with ASC and BMSC at day 7 and in BMSC and chondrocyte cocultures at day 28 compared to respective monocultures. In ASC cocultured with NB, *COL10A1* gene expression was increased compared to OAB cocultures of BMSC and chondrocytes at day 28, while all other OAB coculture conditions revealed no significant difference to NB cocultures (Figure 2D).

Overall, we observed an inhibitory effect of OAB explants on collagen gene expression in co- and tricultures whereas NB explants affected collagen gene expression less.

### Quantification of collagen and proteoglycan synthesis in cell lysates

In further experiments, we studied the influence of OAB explants on collagen (day 28) and GAG/proteoglycan (days 7 and 28) synthesis.

**Figure 3:**
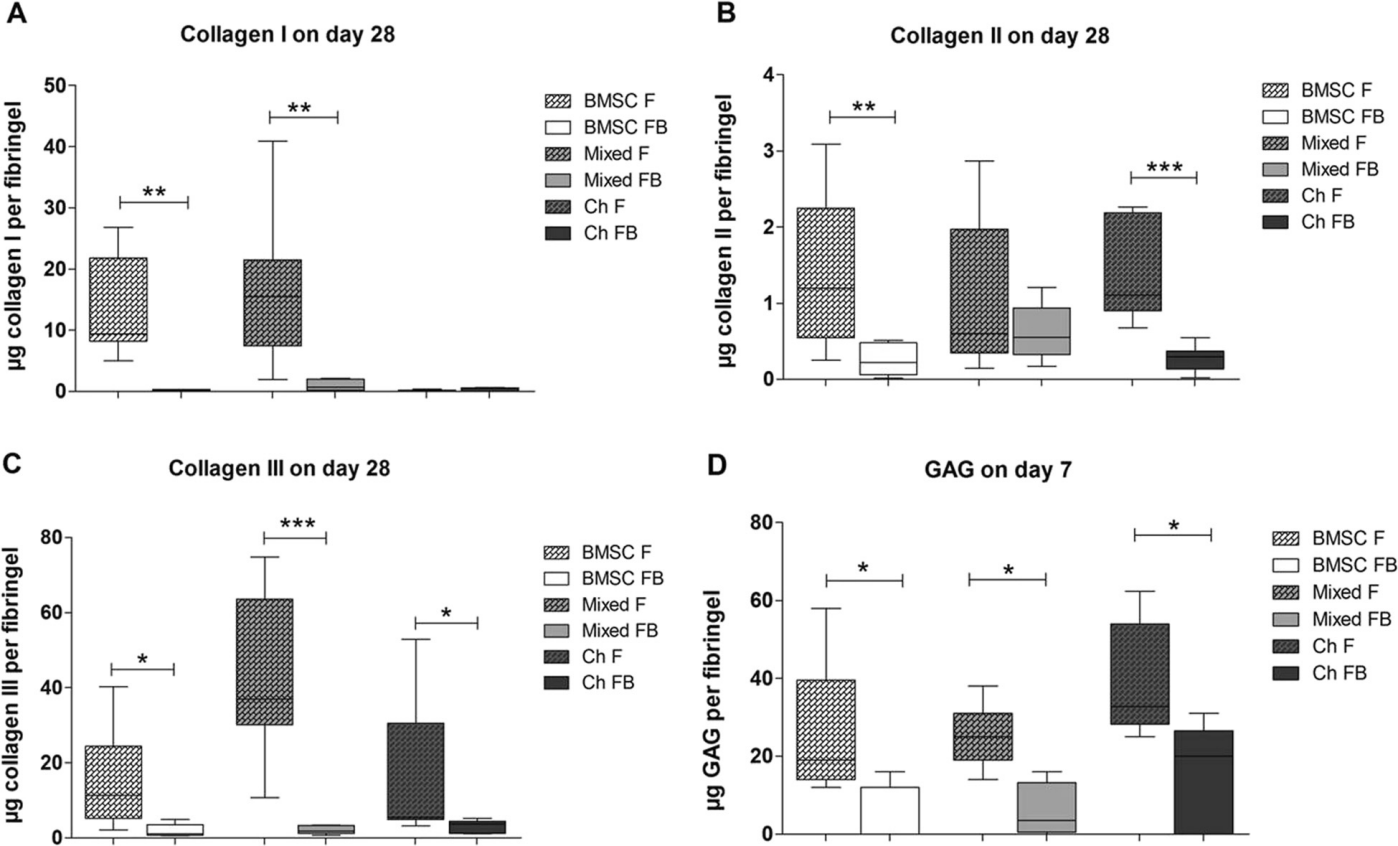
**Quantification of collagens I, II, III and proteoglycans in cell lysates.** Protein synthesis of collagens I **(A)**, II **(B)** and III **(C)** in cell lysates after 28 days of culture in fibrin gels and GAGs **(D)** in cell lysates on day 7. BMSC (white bars), mixed cultures (BMSC and chondrocytes in a ratio of 1:1, grey bars) or chondrocytes (black bars) were embedded in fibrin gel and kept in monoculture (F, bars with pattern) or in co- and triculture with OAB explants (FB, blank bars) in chondrogenic medium. **(A, B)** Collagens I and II were quantified with ELISA, or **(C)** by densitometrically evaluated dot-blot analysis containing a recombinant collagen III standard curve. **(D)** Proteoglycan/GAG concentration in cell lysates was quantified by a DMMB assay including a chondroitin sulfate standard curve. Results are mean with standard deviation (SD). N =5; ^*^ *P* <0.05, ^**^ *P* <0.01, ^***^ *P* <0.001. BMSC, bone marrow-derived mesenchymal stem cells; DMMB, dimethylmethylene blue; F, fibrin gel-embedded monocultures (without subchondral bone explants); FB, fibrin gel-embedded co- and tricultures together with subchondral bone explants; GAG, glycosaminoglycan; OAB, osteoarthritic subchondral bone.

We detected a significantly reduced collagen I production in BMSC cocultures and mixed tricultures in comparison to monocultures. In chondrocytes, collagen I synthesis was below assay detection limit in both culture conditions (Figure 3A). We observed a significantly reduced collagen II production in BMSC and chondrocyte cocultures compared to monocultures while mixed tricultures remained unaffected (Figure 3B). Collagen III production in all three co- and triculture conditions was significantly reduced compared to monocultures (Figure 3C). A dimethylmethylene blue (DMMB) assay demonstrated that all co- and triculture cell lysates had a significantly decreased GAG/proteoglycan content on day 7 (Figure 3D) while on day 28 no significant differences were detected between mono-, co- and tricultures (data not shown).

Overall, we observed an inhibitory effect of OAB on collagen and proteoglycan synthesis in co- and tricultured cells*.*

### Immunofluorescence staining of collagens and histological staining of GAG

Collagen deposition into the ECM of mono-, co- and tricultures was demonstrated by immunofluorescence of cryosections at day 28.

**Figure 4:**
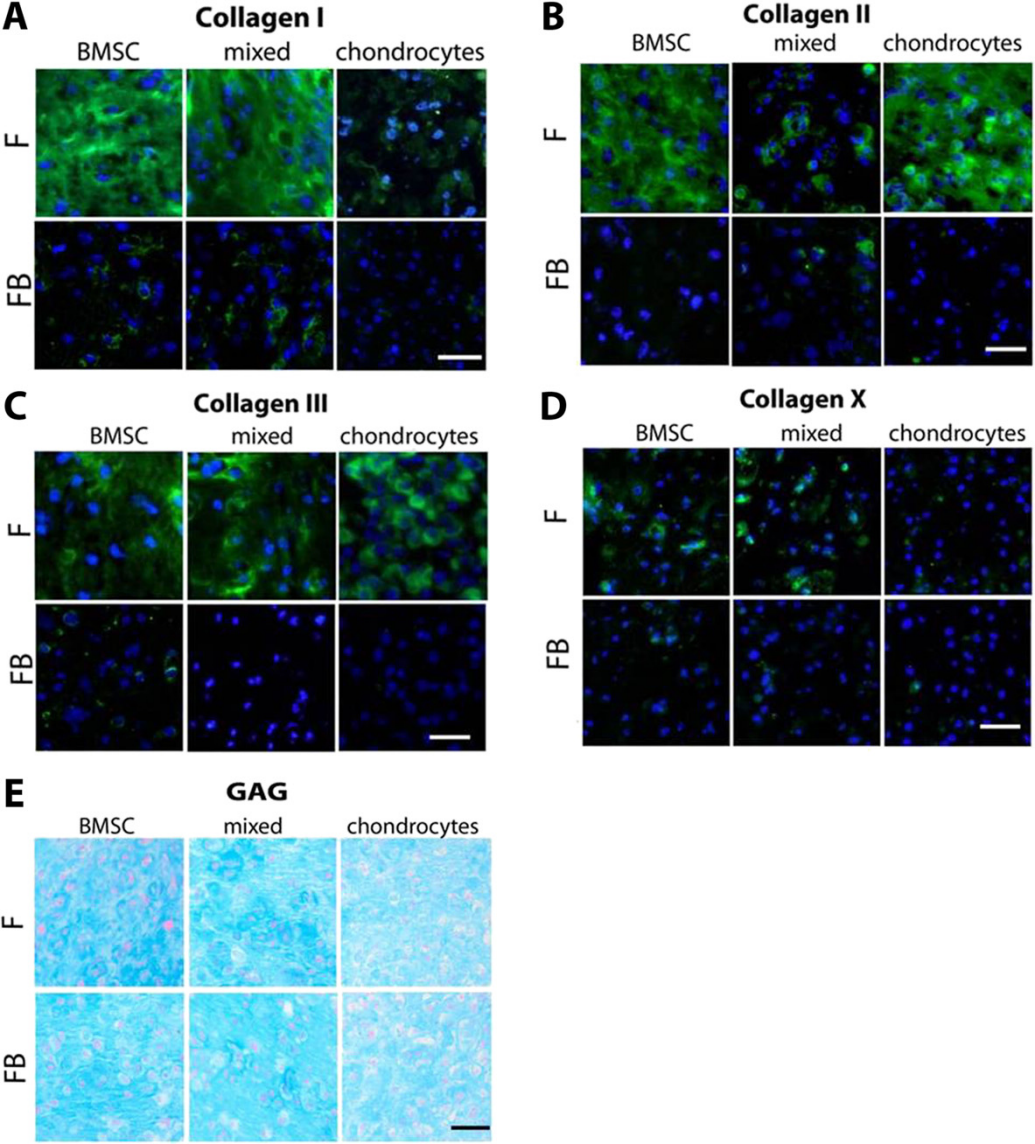
**Immunofluorescence staining of collagens.** Representative immunofluorescence staining of collagens I **(A)**, II **(B)**, III **(C)** and X **(D)** and of Alcian blue **(E)** in fibrin gels of BMSC, mixed cultures (BMSC + chondrocytes 1:1) and chondrocytes after 28 days of co- and triculture with OAB (FB) or monoculture (F) (Scale bar is 100 μm). BMSC, bone marrow-derived mesenchymal stem cells; F, fibrin gel-embedded monocultures (without subchondral bone explants); FB, fibrin gel-embedded co- and tricultures together with subchondral bone explants; OAB, osteoarthritic subchondral bone.

BMSC and mixed monocultures deposited a higher amount of collagen I into their ECM than those co- or tricultured with OAB explants. Staining for collagen I in chondrocytes was poor in both culture conditions (Figure 4A). Staining for collagen II was detected in all monoculture conditions and poor or no staining in co- and tricultures with OAB explants (Figure 4B). We observed similar collagen III staining in all monoculture conditions but only little or no staining in the co- and triculture setups (Figure 4C). A similar trend was detected for collagen X, which was positively stained in BMSC and mixed monocultures but only weak in chondrocyte monocultures and co- and tricultures (Figure 4D). Alcian blue staining was used to detect proteoglycan/aggrecan deposition in the fibrin gel constructs. All culture conditions were uniformly stained blue and showed no differences between monocultures and OAB co- and tricultures (Figure 4E).

Overall, we observed reduced collagen deposition into the ECM in co- and tricultured fibrin gels during the culture period compared to monocultures*.*

### Biomechanical properties

**Figure 5:**
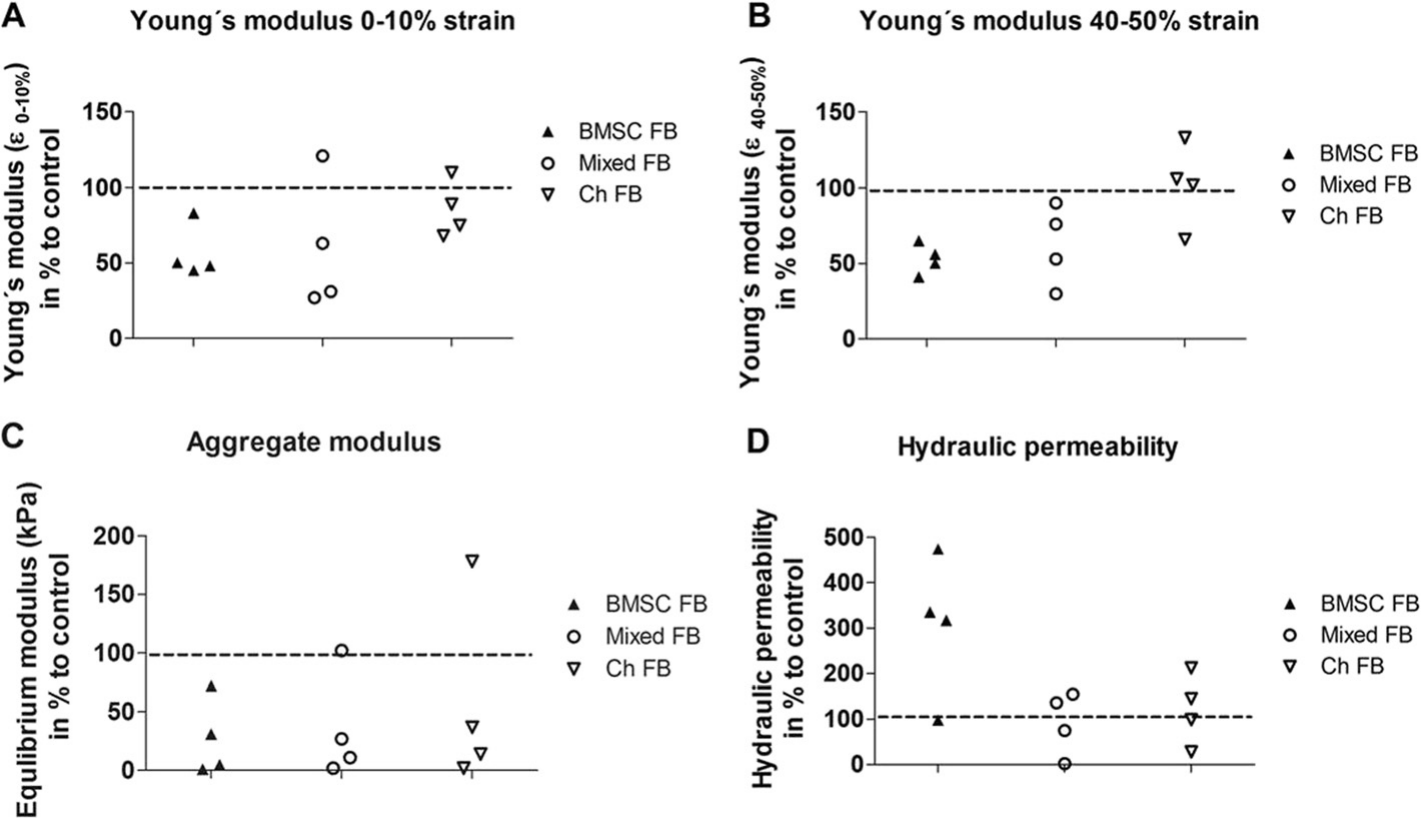
**Determination of biomechanical properties.** Biomechanical properties of the newly formed ECM at day 28 of BMSC, mixed cultures and chondrocytes co- or tricultured with OAB explants were analyzed and calculated to 100% of monocultured controls (dotted lines at 100). Young’s modulus of **(A)** 0 to 10% and **(B)** 40 to 50% strain were determined using unconfined compression. Aggregate modulus at equilibrium **(C)** and hydraulic permeability **(D)** were determined using confined compression performed at 50% compression. N =4. BMSC, bone marrow-derived mesenchymal stem cells; ECM, extracellular matrix; OAB, osteoarthritic subchondral bone.

We determined the integrity and load capacity of newly generated ECM by measuring its biomechanical properties. Unconfined mechanical testing indicated that OAB cocultures exhibited a decrease in Young’s modulus at 0 to 10% strain in four of four samples (BMSC) and three of four samples (mixed tricultures and chondrocytes) (Figure 5A). Young’s modulus at 40 to 50% strain showed a decrease in BMSC and mixed OAB co- and tricultures (four of four) while Young’s modulus of chondrocytes (three of four) was increased (Figure 5B). Aggregate modulus at equilibrium was reduced in all OAB co- and tricultures compared to monocultures (Figure 5C). Further, three of four BMSC OAB coculture samples had a more than threefold hydraulic permeability compared to monocultures while no clear differences in hydraulic permeability were detected for mixed and chondrocyte OAB co- and tricultures compared to monocultures (Figure 5D).

### Quantification of cytokines, bFGF and GAG in culture supernatants

**Figure 6:**
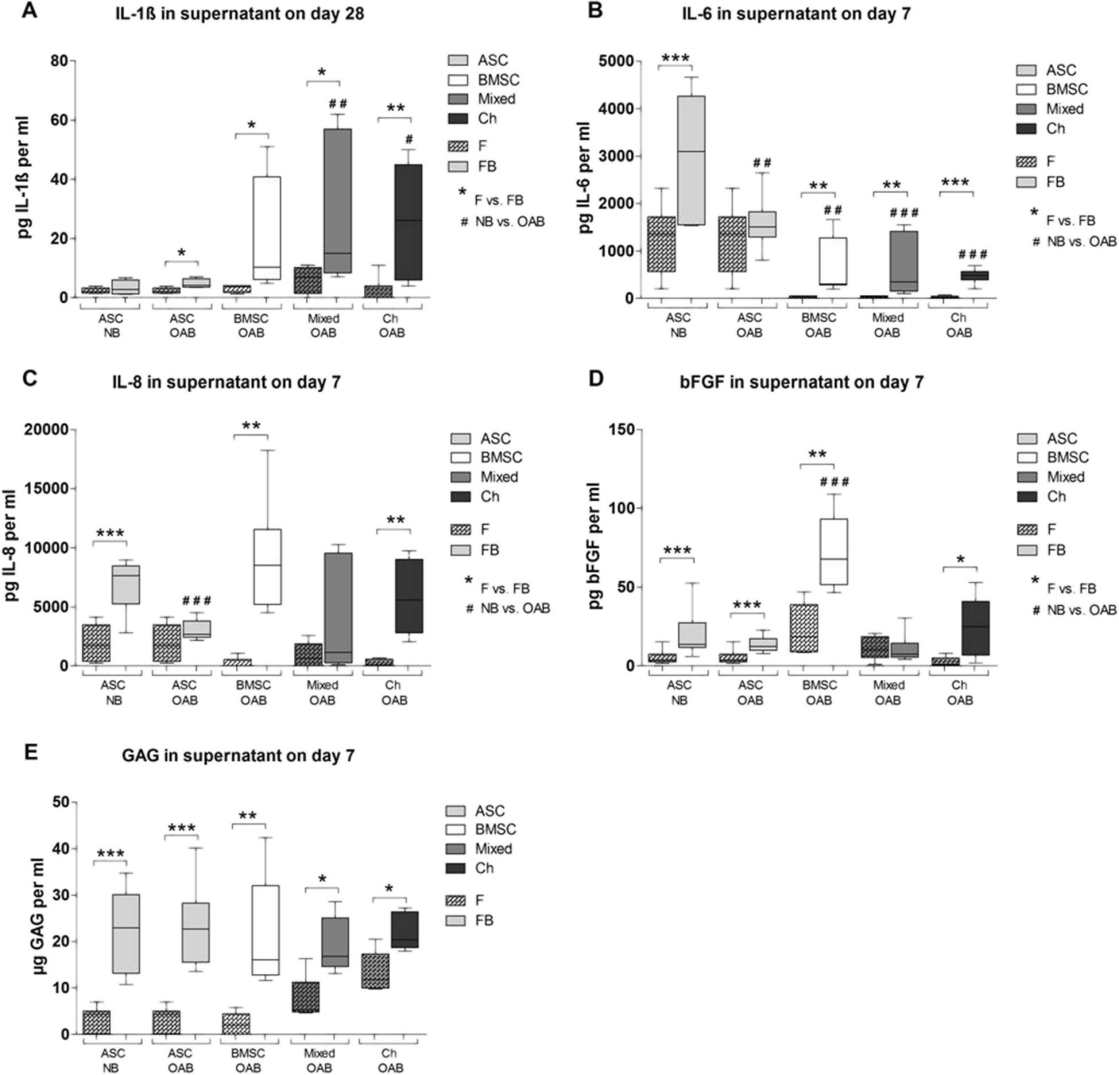
**Quantification of cytokines, bFGF and GAGs in culture supernatants.** Analysis of supernatants at days 7 or 28 of ASC (light grey bars), BMSC (white bars), mixed cultures (BMSC and chondrocytes in a ratio of 1:1, dark grey bars) or chondrocytes (black bars) monocultured (F, bars with pattern) and co- or tricultured with NB or OAB explants (FB, blank bars). Total amounts of cytokines **(A)** IL-1ß, **(B)** IL-6, **(C)** IL-8), **(D)** bFGF released into the supernatant were quantified by antigen-specific ELISAs. Analysis of soluble GAG was carried out using a DMMB-assay containing a chondroitin sulfate standard curve for quantification **(E)**. Stars (^*^) indicate significant differences between mono- and cocultures, hash signs (^#^) indicate significant differences between NB and OAB cocultures. Results are mean with standard deviation (SD). N =6; ^*^ *P* <0.05, ^**^ *P* <0.01, ^***^ *P* <0.001. bFGF, basic fibroblast growth factor; BMSC, bone marrow-derived mesenchymal stem cells; DMMB, dimethylmethylene blue; ELISA, enzyme-linked immunosorbent assay; F, fibrin gel-embedded monocultures (without subchondral bone explants); FB, fibrin gel-embedded co- and tricultures together with subchondral bone explants; GAG, glycosaminoglycan; NB, normal subchondral bone; OAB, osteoarthritic subchondral bone.

In supernatants of ASC, BMSC, mixed cultures and chondrocytes co- or tricultured with OAB explants, we detected significantly more IL-1ß at day 28 compared to monocultures whereas NB did not affect IL-1ß release. In addition, mixed tricultures and chondrocyte cocultures with OAB released more IL-1ß into the culture supernatant compared to ASC cocultures with NB (Figure 6A). We detected a significant higher IL-6 level in NB cocultures of ASC compared to all other OAB co- and triculture regimen at day 7. In addition, all coculture regimens (except for ASC + OAB) released more IL-6 into the culture supernatants compared to monocultures at day 7 (Figure 6B). IL-8 concentrations in supernatant of NB cocultures with ASC and OAB cocultures with BMSC and chondrocytes at day 7 were significantly elevated compared to monocultures. Moreover, IL-8 level in ASC cocultured with OAB was significant lower than in ASC cocultured with NB (Figure 6C). ASC, BMSC and chondrocyte NB and OAB cocultures released more bFGF into supernatants in comparison to monocultures. In supernatants of OAB cocultures with BMSC, significantly higher levels of bFGF were detected compared to ASC cocultures with NB (Figure 6D). We detected a significantly higher soluble GAG (sGAG) level in supernatants of all co- and tricultures at day 7, however, no significant differences between NB and OAB cocultures were detectable (Figure 6E). Analysis of supernatants for total soluble collagen content or soluble fibronectin content did not reveal differences (data not shown).

**Figure 7:**
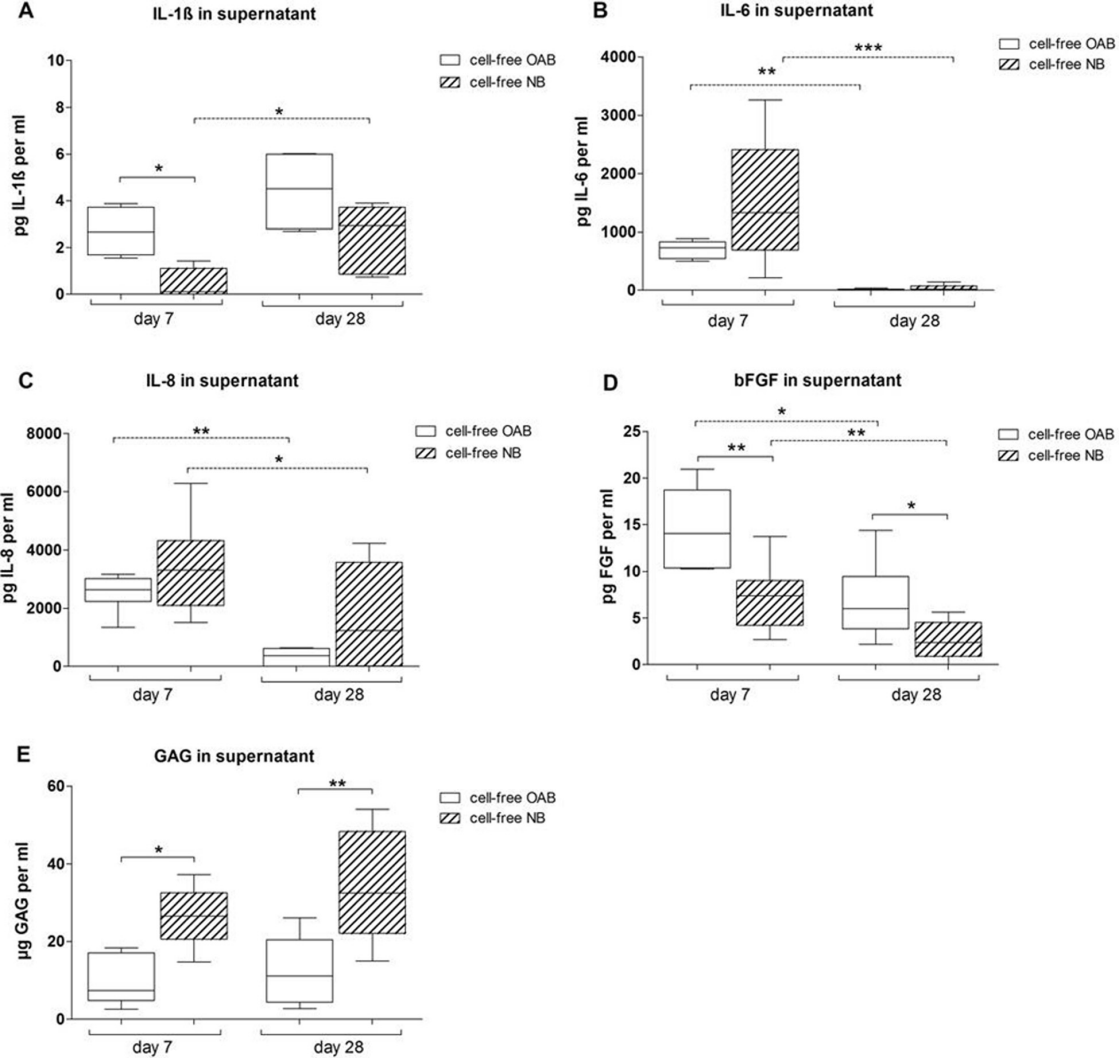
**Quantification of cytokines, bFGF and GAGs in cell-free bone supernatants.** Concentration of **(A)** IL-1ß, **(B)** IL-6 and **(C)** IL-8, **(D)** bFGF and soluble **(E)** GAGs was determined in cell-free OAB explants (white bars) and NB explants (bars with pattern) kept in chondrogenic medium for 7 or 28 days. Results are mean with standard deviation (SD). N =4; ^*^ *P* <0.05; ^**^ *P* <0.01; ^***^ *P* <0.001. bFGF, basic fibroblast growth factor; GAG, glycosaminoglycan; IL, interleukin; NB, normal subchondral bone; OAB, osteoarthritic subchondral bone.

In supernatants of cell-free OAB and NB explants, IL-1ß concentration was in the low pg/ml range but was significantly higher in supernatants of OAB compared to NB at day 7. IL-1ß release increased in NB explant supernatants during culture time (Figure 7A). There was no difference with respect to release of IL-6 and IL-8 between cell-free OAB and NB explants. However, concentration of both cytokines decreased in supernatants during culture time (Figure 7B and C). bFGF concentration in supernatants of cell-free OAB explants was significantly higher than in NB explants at both time points. Additionally, bFGF concentration significantly decreased in supernatants of both, OAB and NB explants from day 7 to day 28 (Figure 7D). Cell-free NB explants revealed at both time points a significantly higher level of GAGs in supernatants compared to OAB explants (Figure 7E).

Overall, we observed an increased level of IL-1ß, IL-6, IL-8, bFGF and GAG in supernatants of co- and tricultures. Cell-free NB or OAB explants released these factors indicating an additive effect of bone tissue and co- or tricultured cells. Additionally, we detected differences in cell-free OAB explants compared to NB culture supernatants for IL-1ß, bFGF and soluble GAG concentrations.

### Stimulation of fibrin gel-embedded monocultures with IL-1ß, IL-6 and IL-8

To determine whether IL-1ß, IL-6 or IL-8 has an effect on gene expression, we stimulated BMSC, mixed and chondrocyte monocultures with these cytokines and determined mRNA expression of *ACAN, MMP2, MMP3* and *MMP13* at culture day 7.

**Figure 8:**
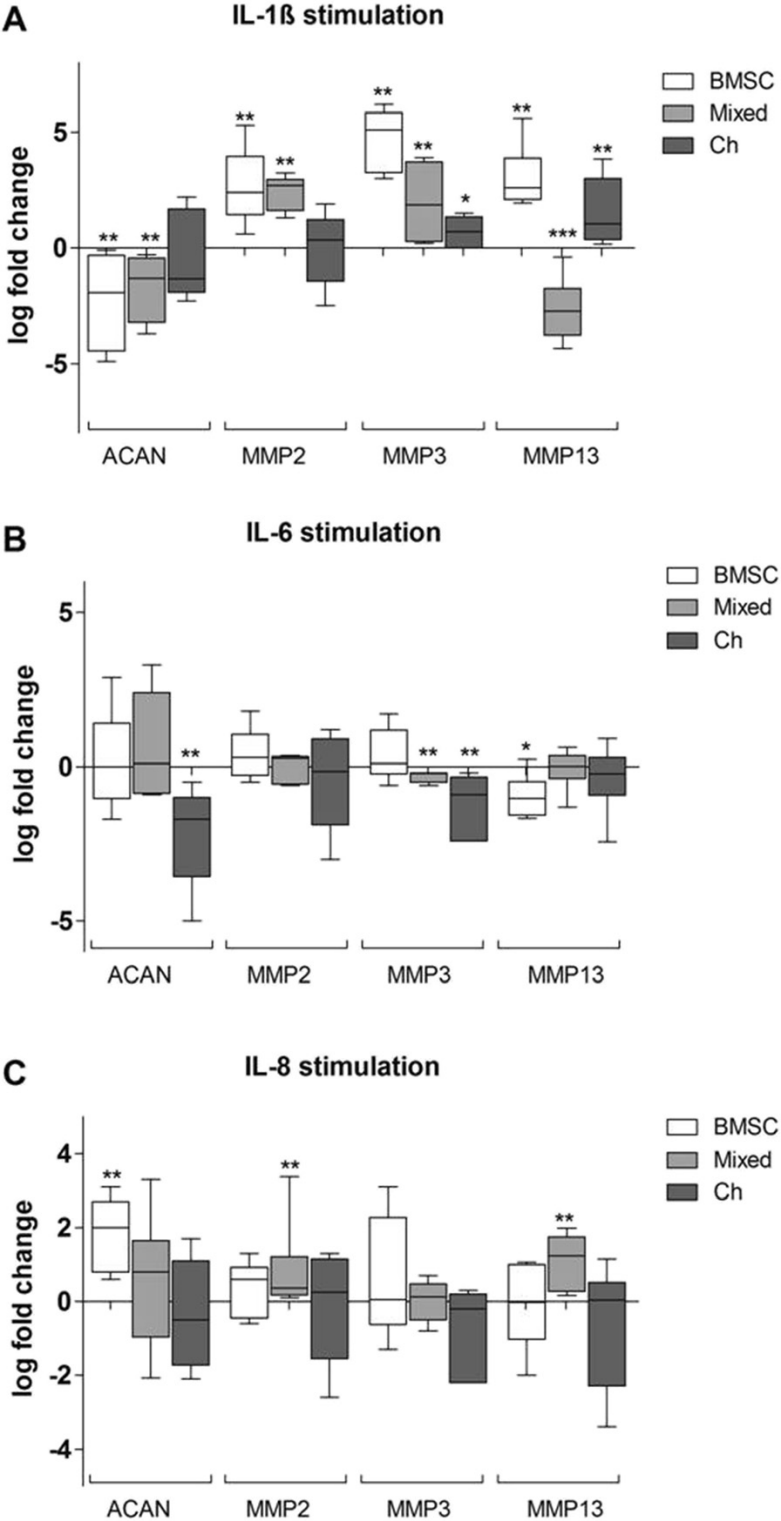
**Stimulation of fibrin gel monocultures with IL-1ß, IL-6 or IL-8 for 7 days.** BMSC (white bars), mixed cultures (BMSC + chondrocytes 1:1, grey bars) or chondrocytes (black bars) embedded in fibrin gel were stimulated for 7 days with 5 ng/ml IL-1ß, 5 ng IL-6 or 10 ng IL-8 in chondrogenic medium-containing dexamethasone and TGF-ß-3. Specimens were analyzed for gene expression of aggrecan (*ACAN*)*,* matrix metalloproteinase (*MMP*)*2*, *MMP3* and *MMP13* at day 7. The log fold change (log_2_) was calculated via a calibrator that represents the unstimulated monocultures and is represented by a line at 0. Results are mean with standard deviation (SD). N =5; ^*^ *P* <0.05, ^**^ *P* <0.01, ^***^ *P* <0.001. BMSC, bone marrow-derived mesenchymal stem cells; IL-1ß, interleukin; IL-6, interleukin-6; IL, interleukin-8; TGF, transforming growth factor.

We observed a significant inhibition of *ACAN* gene expression in IL-1ß-stimulated BMSC and mixed cultures in comparison to unstimulated controls. An increase of *MMP2* gene expression was detected in IL-1ß-stimulated BMSC and mixed cultures while *MMP3* gene expression was induced in all three culture regimens compared to unstimulated controls. We observed a significant induction of *MMP13* gene expression in IL-1ß-stimulated BMSC and chondrocytes whereas *MMP13* was downregulated in mixed cultures (Figure 8A).

IL-6 stimulation reduced *ACAN* gene expression in chondrocyte monocultures compared to unstimulated controls only. *MMP2* gene expression in all three culture conditions remained unchanged. In contrast, *MMP3* expression was downregulated in mixed cultures and chondrocytes in comparison to unstimulated monocultures. IL-6 stimulation reduced *MMP13* gene expression in BMSC monocultures compared to unstimulated controls (Figure 8B).

Stimulation with IL-8 induced *ACAN* gene expression in BMSC. *MMP2* gene expression was significantly increased in mixed cultures while *MMP3* gene expression in all other culture conditions remained unaffected. Stimulation with IL-8 increased *MMP13* gene expression in mixed cultures (Figure 8C).

Overall, we observed a mostly inhibitory effect of IL-1ß and IL-6 on gene expression of *ACAN. MMP2*, *MMP3* and *MMP13* gene expression was induced mainly by IL-1ß while IL-6 has no effect on *MMP2* expression and rather downregulates *MMP3* and *MMP13* gene expression. IL-8 stimulation has also only little effect on gene expression except for a partly induction of *ACAN*, *MMP2* and *MMP13*.

### Collagen gene expression of BMSC supplemented with conditioned medium

Fibrin gel-embedded BMSC monocultures supplemented with OAB conditioned medium (CM) were analyzed for gene expression of collagens.

At day 7, no differences between BMSC monocultures with and without conditioned medium were observed concerning gene expression of all analyzed collagens. Notably, at day 28 BMSC incubated with CM from OAB revealed a significantly reduced gene expression of all analyzed collagens compared to nonconditioned medium (Figure S3A-D in Additional file 3).

In addition, we detected a significant upregulation of *COL2A1* and *COL10A1* gene expression from day 7 to day 28 in nonconditioned cultures whereas *COL3A1* was significantly downregulated. Inhibition of *COL2A1* and *COL3A1* gene expression at day 28 compared to day 7 was also detected in cultures with CM (Figure S3B-D in Additional file 3).

Overall, we observed a clear inhibitory effect of OAB CM on gene expression of all collagens at day 28.

Table S1 in Additional file 4 summarizes all *P* values indicated in Figures 2, 3, 6, 7, 8 and Figure S3 in Additional file 3.

## Discussion

A so far unmet challenge in the treatment of OA-affected joints is to replace the degraded articular cartilage and subchondral bone matrix with healthy cartilage/bone tissue in order to restore impaired joint function and to prevent or delay total joint replacement by artificial endoprothesis. Cell-based treatment such as autologous chondrocyte implantation (ACI), microfracturing of trauma-induced focal chondral or osteochondral lesions in younger patients prior to establishment of posttraumatic OA is established and leads in general to production of cartilage- and subchondral bone-like replacement tissue [22,23]. However, there are conflicting data about long-term stability and functionality of specifically cartilage repair tissue as the composition resembles fibrocartilage rather than hyaline cartilage [24,25]. In addition, for older patients or those affected with OA, these treatment options are not successful due to failure or inferior osteochondral defect healing [26]. The reasons for poor healing and repair outcome of cell-based therapies, especially in OA joints, are not comprehensively understood. One likely reason are profoundly altered microenvironmental cues from the surrounding subchondral bone tissue.

In the present study, we analyzed the impact of OAB on chondrogenic differentiation of BMSC, the phenotype of differentiated chondrocytes and matrix-forming capacity. We investigated the influence of OAB joints on BMSC and chondrocytes assuming that factors produced by OA osteoblasts, osteocytes or osteoclasts can modulate metabolic properties of these cells. It is striking that OAB inhibits gene expression and protein production of collagens I, II, III and X in most co- and triculture setups compared to monocultures. This decrease of collagens is not due to increased degradation of existing collagens known to have an *in vivo* half-life of more than 100 years [27] as a hydroxyproline assay did not reveal increased level of soluble collagens in culture supernatants, indicating that reduced biosynthesis is the major reason for reduced collagen matrix deposition, which in turn causes diminished accumulation of collagen protein in the ECM during the culture time. Since the half-life of collagen mRNA is often only several hours [28], differences in collagen protein level at the culture endpoint are not mandatorily mirrored at mRNA level assessed at the same time.

As synthesis of all investigated collagen types was suppressed in the presence of OAB (albeit collagen X production was not quantified), no effect specifically on chondrogenic differentiation of BMSC by subchondral bone can be postulated but a general effect on synthesis of matrix macromolecules. Similar inhibitory effects of OA cartilage explants on the production of collagens were described in a recent study by Leyh *et al*. [29].

When repeating key experiments (gene expression studies and cytokine release) with ASC and NB explants we were unable to detect alterations in gene expression of collagens I and II in coculture with NB suggesting that inhibition, respective alteration of these collagens in ASC cocultures with OAB is related to factors specific for OA.

The interaction between subchondral bone cells and chondrocytes on the molecular level has not been well understood until now. But there are likely important regulatory events after cartilage injury initiated by subchondral bone cells. In line with our observations, Jiang *et al*. revealed a suppression of specific markers in cocultures of chondrocytes and osteoblasts like diminished GAG and collagen II deposition or mineralization of ECM [30]. Coculturing of OA chondrocytes with OA osteoblasts induces inhibition of aggrecan production and a concomitant significant increase in *MMP13* synthesis. The cocultured osteoblasts also decrease *COL2A1*, *SOX9* and *PTHrP/PTH-receptor* gene expression in the chondrocytes [31,32]. In addition to their data, we observed that in cocultures with subchondral bone (OAB and NB) and in cultures with OAB CM gene expression of *COL10A1* was strongly inhibited compared to monocultures. A specific collagen X-suppressing effect by paracrine coculture of BMSC with healthy articular cartilage was reported earlier from our group and thus appears not to be restricted to bone but to as yet unidentified soluble factor(s) that are common to both tissue types and that are OA independent [19].

Notably, besides reduced synthesis of GAGs in cocultured cell lysates at early time points, we also detected increased sGAGs in supernatants of cocultures. However, as OAB and NB cultured without cells also released sGAGs into the supernatant, we believe that we observed an additive effect of cells and explants and not a true increase in degradation of proteoglycans. This is underscored by our observations with ASC cocultures and also by Alcian blue histology, which did not reveal differences between culture conditions at the end of culture (day 28) but at the beginning and by biomechanical properties with respect to permeability and fluid exchange (see below). Therefore, we assume no increase in degradation of proteoglycans and suggest that early inhibition of GAG synthesis is compensated in cocultures during the time line of culture.

We observed an increased release of bFGF in cocultures of BMSC, ASC and chondrocytes with OAB and in cocultures of ASC with NB explants at the early phase of culture, which is in line with a previous study [33]. As we found also release of bFGF from OAB and NB explants cultured without cells, we assume that this adds to the bFGF detected in cocultures. bFGF and other growth factors might be released together with the GAGs they were bound to during pathophysiological situations, that is after tissue injury or during chronic inflammation [34]. Moreover, NB explants released less bFGF compared to OAB explants indicating a true induction of bFGF release from ASC specifically in the presence of normal bone cells.

Reduced synthesis of matrix components is likely to influence mechanical properties and stability of the resulting matrix. Most important for a successful osteochondral repair is the mechanical stability of the new tissue. The ECM of cartilage consists primarily of three molecules: water, type II collagen and the proteoglycan aggrecan [35,36]. Under strain, load is first carried by water, which is displaced from the tissue and leaves the matrix, which carries the load after water has gone from the tissue. Therefore both are physiologically important: water content and its retention in the extrafibrillar matrix together with stability of the fibrillar network. We observed that Young’s modulus was reduced in OAB cocultures of BMSC and mixed cultures whereas aggregate modulus was reduced in all cocultures. Hydraulic permeability showed no clear trend for OAB tricultures and chondrocyte OAB cocultures. Erickson *et al*. showed that in every case, BMSC-laden constructs possessed mechanical properties significantly lower than those of chondrocyte-seeded hydrogel constructs [37], which is in line with our findings. Our results suggest that coculture with subchondral bone leads to reduced loading capacity of matrix, with increased permeability at least for the BMSC coculture. In contrast, OAB-cocultured chondrocytes showed a trend to increased Young’s modulus (depending on strain applied), while hydraulic permeability and aggregate modulus also remained unchanged by subchondral bone coculture. OAB-cocultured chondrocytes seem to produce a matrix with mechanical properties that resemble monocultured OA chondrocytes - which does not mean that it is comparable to an ECM synthesized by healthy chondrocytes. Taken together, our data suggest that coculture with OAB impairs mechanical matrix properties with respect to the newly formed fibrillar collagenous network and not of the proteoglycan rich extrafibrillar matrix. This is in line with a previous study from our group, which indicates a distinct modulatory influence of OA cartilage explants that affects the collagen composition of the *de novo*-produced ECM from co- and tri-cultured cells and leads to impaired mechanical and biochemical properties of the matrix because of an altered fibrillar network [29]*.*

Although many studies have examined effects of factors released from cartilage and chondrocytes on chondrogenesis, only few have focused on the influence of proinflammatory cytokines from subchondral bone on the chondrogenic differentiation and matrix-forming capacity of BMSC [38,32]. In the current study, we analyzed proinflammatory cytokines like IL-1ß, IL-6 and IL-8, which are known to be released from BMSC and OA chondrocytes and are considered to contribute to OA pathogenesis [39,40]. We discovered that coculture with OAB truly induced the concentration of these cytokines/chemokines in culture supernatants in all culture conditions. This was most prevalent in the early phase of the culture except for IL-1ß, which remained induced throughout the culture time. Notably, comparison of inflammatory factors released by OAB and NB explants in cell-free and coculture experiments revealed differences. First, cell-free NB explants released less IL-1ß and bFGF but more GAGs than OAB and - importantly - NB did not induce IL-1ß release in coculture. Second, IL-6 and (partly) IL-8 release in cocultures with NB explants was higher than release from OAB cocultures. Presumably, these metabolic differences result from a differently composed ECM of bone tissue from younger trauma patients, including increased osteoblast numbers and activity resulting in a higher bone-forming capacity compared to older OAB [41,42]. We suggest that these cytokines in part mediate suppression of collagen and GAG synthesis and induce GAG release from proteoglycans, which would be in line with data from literature [32,43]. To prove our hypothesis, we stimulated monocultures of BMSC, mixed cells and chondrocytes - embedded in fibrin gel - with IL-1ß, IL-6 or IL-8. We observed that stimulation with IL-1ß in general induced *MMP2, MMP3* and *MMP13* gene expression, which all are important players with respect to matrix degradation. Additionally, IL-1ß-stimulation of BMSC and of mixed cultures leads to a significant downregulation of *ACAN* gene expression, which is in line with most recent literature [39], however, Salter *et al*. reported involvement of IL-1ß in a chondroprotective signaling cascade, which is defined by increasing *ACAN* mRNA in chondrocytes [44]. In concert with IL-1ß, IL-6 inhibits proteoglycan production and can in addition amplify IL-1ß effects [39]. We observed that IL-6 effects on *ACAN* gene expression were less pronounced as after IL-1ß stimulation. *MMP2* expression was not affected while *MMP3* and *MMP13* expression were mostly downregulated after IL-6 stimulation. This is in line with studies that predict that IL-6 might have also a beneficial role in OA pathology. OA chondrocytes produce more IL-6 during cartilage regeneration than healthy chondrocytes. A recent study of Tsuchida *et al*. revealed a modest anabolic role for IL-6 in cartilage matrix regeneration demonstrating that chondrocytes produce high amounts of IL-6, which is released into the synovial fluid and has possible beneficial effects on cartilage regeneration [45]. It appears that chondrocytes react more sensitive to OA conditions as we observed no differences in IL-6 and IL-8 level in OAB versus NB bone explant supernatants.

We demonstrated inductive effects of IL-8 on gene expression of mixed cultures for *MMP2* and *MMP13* and of BMSC for *ACAN*, while chondrocytes did not respond. IL-8 initiates chemotaxis in cells and leads to cell migration aligned with *MMP* upregulation. This could be interpreted as a trial for cartilage repair with local BMSC and suggests, that IL-8 is required to initiate tissue repair *in vivo* [46]. Notably, chondrocytes seemed to be not affected by IL-8 stimulation possibly, because OA chondrocytes are less sensitive to cytokine stimulation than normal cells [47].

In addition, we wanted to answer the question if differentiated chondrocytes, which are in close cell-to-cell contact with undifferentiated BMSC ameliorate or augment inhibitory effects of subchondral bone explants on BMSC. Overall, we observed that collagen gene expression was mostly unaltered while protein expression and biomechanical properties of OAB tricultured mixed populations resemble rather OAB cocultured BMSC than chondrocytes. We thus suggest that chondrocytes in the mixed cultures do not alter BMSC metabolism in tricultures with subchondral bone explants and that response of mixed cultures to factors from OAB resemble rather that of cocultured BMSC than that of cocultured chondrocytes.

In order to include a ‘reference’ for BMSC that were all obtained from OA-affected donors, we repeated key experiments with ASC which served as ‘healthy’ controls. We did not find striking differences between both cell types with respect to influence of coculture on collagen gene expression and cytokine, bFGF and GAG release. Thus we suggest that tissue source (ASC versus BMSC) and age and disease status (OA versus non-OA) of cell donors do not affect matrix-forming capacity and chondrogenic differentiation at least under *in vitro* conditions.

In order to decide if observed effects are related to OA, we have repeated key experiments with subchondral bone from trauma patients. We observed differences in release of IL-1ß, bFGF and GAG between OAB and NB cell-free explants and also with respect to gene expression and cytokine release in OAB versus NB cocultures. However, a limitation of our study is caused by the control group, which consists of trauma patients who have a lower mean age than those individuals with OA included into the study group. Therefore, differences in cytokine release as we have observed between NB and OAB might result not only from OA pathogenesis but also from different metabolic activities due to the age of the bone donor. In the current study, it is thus not possible to assign these metabolic alterations specifically to OA as age of bone donors might have also an impact.

## Conclusions

We observed elevated soluble factors in coculture supernatants compared to monocultures in parallel to a metabolic shift in cocultured BMSC and OA chondrocytes, which affects synthesis of collagens and proteoglycans. This reduced biosynthesis of structural macromolecules alters composition of the fibrillar collagen networks of the ECM and impairs mechanical stability and integrity of the newly formed matrix. However, impaired production of proteoglycans appears to be compensated during culture time and thus not to be responsible for inferior mechanical properties of the ECM.

We suggest that these effects are at least partly mediated by IL-1ß, bFGF and IL-6, which are increased in coculture supernatants while IL-8 effects are less pronounced with respect to investigated parameters.

Our data imply that implanting BMSC or chondrocytes into subchondral bone lesions or onto the surface of denuded OA bone will not result in formation or regeneration of a well functional cartilage-like matrix. Thus before trying to restore eroded cartilage tissue in an OA-affected joint by a cell-based approach, release of proinflammatory cytokines and additional soluble factors as GAGs and growth factors from surrounding tissues has to be strictly controlled.

## Additional files

Additional file 1: Figure S2. FACS analysis of BMSC surface markers and staining of adipogenic and osteogenic differentiated BMSC. **(A)** Isolated plastic adherent BMSC in passage 3 were analyzed by flow cytometry using specific antibodies against BMSC negative markers CD 19 and CD 34 and BMSC positive markers CD 44 and CD 105 (red line). Specificity of epitope staining was proofed using an isotype control (black line). **(B)** BMSC were stained with oil red for oil drops incorporated during adipogenic differentiation (upper row) and with alizarin red for matrix mineralization during osteogenic differentiation (lower row). Additional file 2: Figure S1. Vitality of fibrin gel-embedded cells and coculture setups. Vitality of **(A)** BMSC, **(B)** mixed cultures (BMSC and chondrocytes in a ratio of 1:1) and **(C)** chondrocytes was determined in monocultures (F) and co- or tricultures with OAB (FB) kept in chondrogenic medium. Content of LDH was quantified in the supernatant of days 7, 14, 21 and 28 and compared to assay controls (high control = all cells in fibrin gels were lysed; low control = spontaneous cell death of an equivalent cell amount in monolayer). Due to high interexperimental variability, we have calculated the raw data as a percentage of control per individual experiment. Results are mean with standard variation (SD). N =4; ^*^ *P* <0.05, ^**^ *P* <0.01, ^***^ *P* <0.001. Additional file 3: Figure S3. Quantification of gene expression in BMSC monocultures stimulated with OA bone explants conditioned medium. Gene expression level of **(A)** *COL1A1,* **(B)** *COL2A1,* **(C)** *COL3A1* and **(D)** *COL10A1* were determined in monocultures supplemented with OAB-conditioned medium using plasmid standard curves. BMSC monoculture with fresh medium (F, bars with pattern) and monoculture of BMSC supplemented with OAB conditioned chondrogenic medium (F + CM, blank bars) were analyzed after 7 or 28 days. Due to high interexperimental variability we have calculated the raw data as a percentage of highest cDNA copy number per individual experiment. Solid lines indicate significant differences between culture conditions, dotted lines indicate significant differences between culture time points (days 7 and 28). N =4; ^*^ *P* <0.05, ^**^ *P* <0.01, ^***^ *P* <0.001. Additional file 4: Table S1. Statistical data (*P* values). *P* values of: **(A)** Cocultures vs. monocultures and normal vs. OA subchondral bone cocultures. *COL1A1*, *COL2A1*, *COL3A1* and *COL10A1* gene expression determined in ASC, BMSC, mixed cultures or chondrocytes kept as monocultures (F) or as co- and tricultures with osteoarthritic (OA FB) or normal subchondral bone explants (NB FB) at day 7 and day 28 (corresponding to Figure 2). **(B)** Cocultures vs. monocultures. Collagen protein (day 28) and proteoglycan (day 7) quantification in cell lysates from BMSC, mixed cultures or chondrocytes kept as monocultures (F) or as co- and tricultures with OA subchondral bone explants (FB) (corresponding to Figure 3).

## Abbreviations

ACAN: aggrecan gene
ACI: autologous chondrocyte implantation
ASC: adipose-derived stem cells
bFGF: basic fibroblast growth factor
BMSC: bone marrow-derived mesenchymal stem cells
BSA: bovine serum albumin
CM: conditioned medium
Col10A1: gene collagen X
COL1A1: gene collagen I
COL2A1: gene collagen II
COL3A1: gene collagen III
DMEM: Dulbecco’s modified Eagle’s medium
DMMB: dimethylmethylene blue
ECM: extracellular matrix
ELISA: enzyme-linked immunosorbent assay
FCS: fetal calf serum
GAG: glycosaminoglycan
IL-1ß: interleukin 1ß
IL-6: interleukin 6
IL-8: interleukin 8
LDH: lactate dehydrogenase
MMP: matrix metalloproteinase
NB: normal subchondral bone
OA: osteoarthritis
OAB: OA subchondral bone
PBS: phosphate-buffered saline
PFA: paraformaldehyde
TBS: Tris-buffered saline
TEP: total knee replacements
PTHrP/PTH: parathyroid hormone-related protein/parathyroid hormone receptor
TGF-ß: transforming growth factor ß
