# Supplementary figures and images for: Subchondral bone influences chondrogenic differentiation and collagen production of human bone marrow-derived mesenchymal stem cells and articular chondrocytes
Source: Arthritis Res Ther. 2014 Oct 7;16(5):453. doi: 10.1186/s13075-014-0453-9 (PMC4209060; doi:10.1186/s13075-014-0453-9)

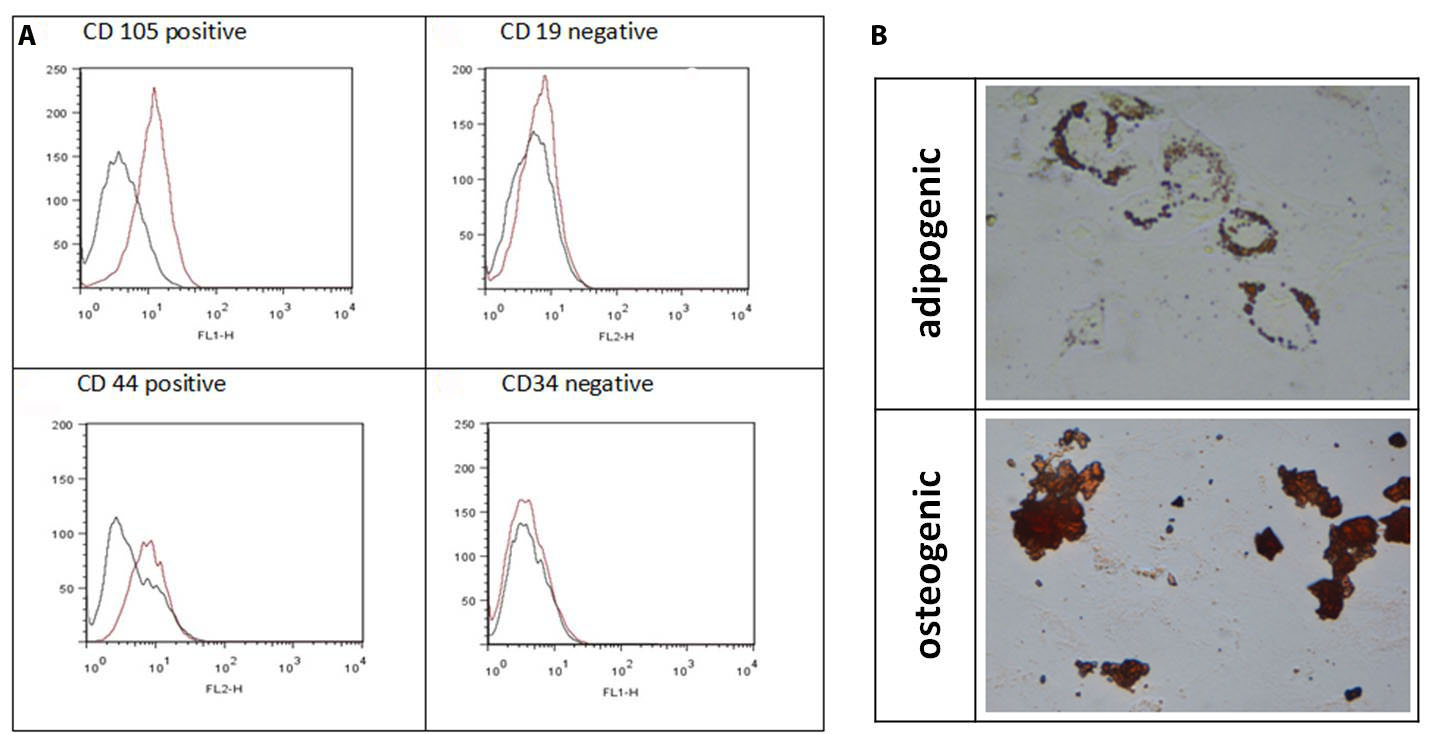

Supplement: Additional file 1: Figure S2. — FACS analysis of BMSC surface markers and staining of adipogenic and osteogenic differentiated BMSC. (A) Isolated plastic adherent BMSC in passage 3 were analyzed by flow cytometry using specific antibodies against BMSC negative markers CD 19 and CD 34 and BMSC positive markers CD 44 and CD 105 (red line). Specificity of epitope staining was proofed using an isotype control (black line). (B) BMSC were stained with oil red for oil drops incorporated during adipogenic differentiation (upper row) and with alizarin red for matrix mineralization during osteogenic differentiation (lower row). [file 13075_2014_453_MOESM1_ESM.jpeg]

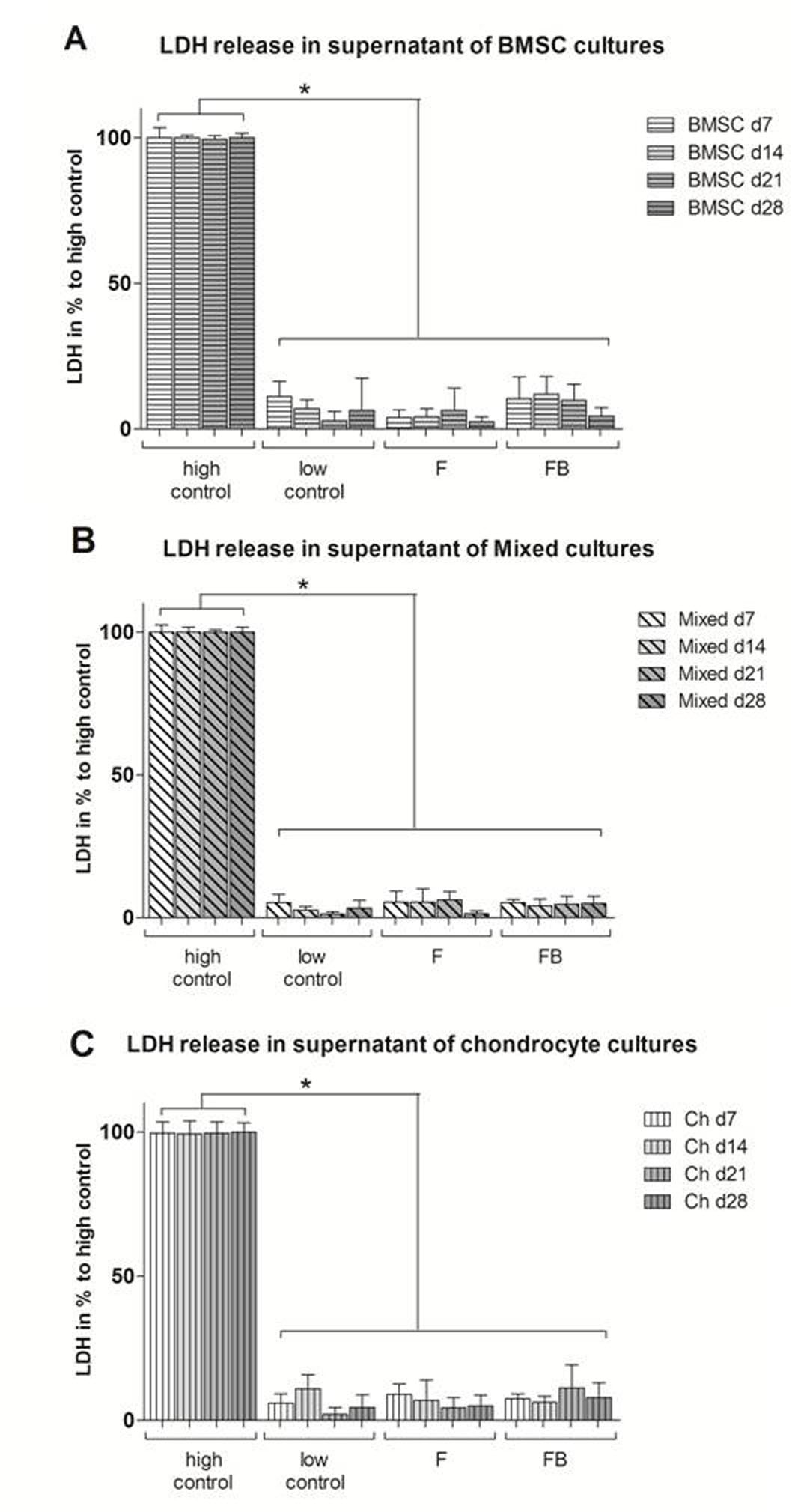

Supplement: Additional file 2: Figure S1. — Vitality of fibrin gel-embedded cells and coculture setups. Vitality of (A) BMSC, (B) mixed cultures (BMSC and chondrocytes in a ratio of 1:1) and (C) chondrocytes was determined in monocultures (F) and co- or tricultures with OAB (FB) kept in chondrogenic medium. Content of LDH was quantified in the supernatant of days 7, 14, 21 and 28 and compared to assay controls (high control = all cells in fibrin gels were lysed; low control = spontaneous cell death of an equivalent cell amount in monolayer). Due to high interexperimental variability, we have calculated the raw data as a percentage of control per individual experiment. Results are mean with standard variation (SD). N =4; * P <0.05, ** P <0.01, *** P <0.001. [file 13075_2014_453_MOESM2_ESM.jpeg]

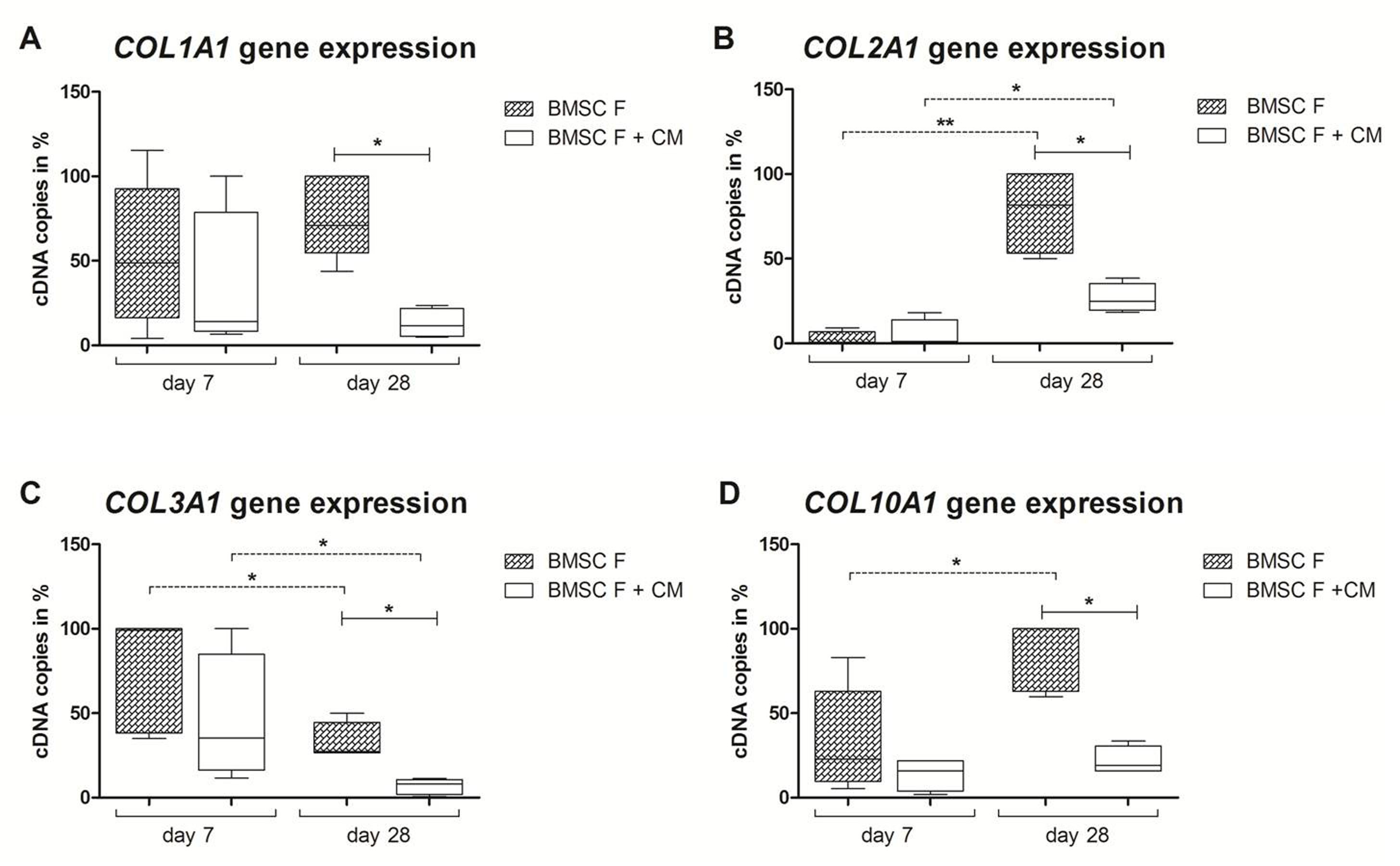

Supplement: Additional file 3: Figure S3. — Quantification of gene expression in BMSC monocultures stimulated with OA bone explants conditioned medium. Gene expression level of (A) COL1A1, (B) COL2A1, (C) COL3A1 and (D) COL10A1 were determined in monocultures supplemented with OAB-conditioned medium using plasmid standard curves. BMSC monoculture with fresh medium (F, bars with pattern) and monoculture of BMSC supplemented with OAB conditioned chondrogenic medium (F + CM, blank bars) were analyzed after 7 or 28 days. Due to high interexperimental variability we have calculated the raw data as a percentage of highest cDNA copy number per individual experiment. Solid lines indicate significant differences between culture conditions, dotted lines indicate significant differences between culture time points (days 7 and 28). N =4; * P <0.05, ** P <0.01, *** P <0.001. [file 13075_2014_453_MOESM3_ESM.jpeg]
