# Supplementary material for: Subchondral bone influences chondrogenic differentiation and collagen production of human bone marrow-derived mesenchymal stem cells and articular chondrocytes
Source: Arthritis Res Ther. 2014 Oct 7;16(5):453. doi: 10.1186/s13075-014-0453-9 (PMC4209060; doi:10.1186/s13075-014-0453-9)
Supplement: Additional file 4: Table S1. — Statistical data (P values). P values of: (A) Cocultures vs. monocultures and normal vs. OA subchondral bone cocultures. COL1A1, COL2A1, COL3A1 and COL10A1 gene expression determined in ASC, BMSC, mixed cultures or chondrocytes kept as monocultures (F) or as co- and tricultures with osteoarthritic (OA FB) or normal subchondral bone explants (NB FB) at day 7 and day 28 (corresponding to Figure 2). (B) Cocultures vs. monocultures. Collagen protein (day 28) and proteoglycan (day 7) quantification in cell lysates from BMSC, mixed cultures or chondrocytes kept as monocultures (F) or as co- and tricultures with OA subchondral bone explants (FB) (corresponding to Figure 3). [file 13075_2014_453_MOESM4_ESM.doc]

**Table 1: Statistical data (p-values)**

**A) Quantification of gene expression with qPCR (Fig. 2)**

| **Col I coculture** | ASC NB day 7 | ASC NB day 28 | ASC OA day 7 | ASC OA day 28 | BMSC OA day 7 | BMSC OA day 28 | Mixed OA day 7 | Mixed OA day 28 | Ch OA day 7 | Ch OA day 28 |
| --- | --- | --- | --- | --- | --- | --- | --- | --- | --- | --- |
| **vs. monoculture** | 0.9009 | 0.2622 | 0.0109* | 0.2622 | 0.0179 * | 0.6723 | 0.0179* | 0.1388 | 0.9266 | 0.4070 |
| **Col II coculture** | ASC NB day 7 | ASC NB day 28 | ASC OA day 7 | ASC OA day 28 | BMSC OA day 7 | BMSC OA day 28 | Mixed OA day 7 | Mixed OA day 28 | Ch OA day 7 | Ch OA day 28 |
| **vs. monoculture** | 0.9009 | 0.2622 | 0.9264 | 0.0112 * | 0.2622 | 0.0179 * | 0.2622 | 0.9009 | 0.4070 | 0.6723 |
| **Col III coculture** | ASC NB day 7 | ASC NB day 28 | ASC OA day 7 | ASC OA day 28 | BMSC OA day 7 | BMSC OA day 28 | Mixed OA day 7 | Mixed OA day 28 | Ch OA day 7 | Ch OA day 28 |
| **vs. monoculture** | 0.0179 * | 0.3832 | 0.0107 * | 0.2622 | 0.0179 * | 0.6723 | 0.0179 * | 0.0179 * | 0.0179 * | 0.0179 * |
| **Col X coculture** | ASC NB day 7 | ASC NB day 28 | ASC OA day 7 | ASC OA day 28 | BMSC OA day 7 | BMSC OA day 28 | Mixed OA day 7 | Mixed OA day 28 | Ch OA day 7 | Ch OA day 28 |
| **vs. monoculture** | 0.9009 | 0.0175 * | 0.0109 * | 0.0800 | 0.0179 * | 0.0112 * | 0.9009 | 0.9009 | 0.6723 | 0.0179 * |

| **Col I coculture** | ASC OA day 7 | ASC OA day 28 | BMSC OA day 7 | BMSC OA day 28 | Mixed OA day 7 | Mixed OA day 28 | Ch OA day 7 | Ch OA day 28 |
| --- | --- | --- | --- | --- | --- | --- | --- | --- |
| **vs. ASC NB FB** | 0.2622 | 0.1143 | 0.4857 | 0.9009 | 0.6857 | 0.0317 ^#^ | 0.6095 | 0.0667 |
| **Col II coculture** | ASC OA day 7 | ASC OA day 28 | BMSC OA day 7 | BMSC OA day 28 | Mixed OA day 7 | Mixed OA day 28 | Ch OA day 7 | Ch OA day 28 |
| **vs. ASC NB FB** | 0.9141 | 0.4127 | 0.4857 | 0.0571 | 0.4875 | 0.1143 | 0.9141 | 0.0635 |
| **Col III coculture** | ASC OA day 7 | ASC OA day 28 | BMSC OA day 7 | BMSC OA day 28 | Mixed OA day 7 | Mixed OA day 28 | Ch OA day 7 | Ch OA day 28 |
| **vs. ASC NB FB** | 0.2602 | 0.6857 | 0.9017 | 0.9021 | 0.0639 | 0.1143 | 0.3429 | 0.0571 |
| **Col X coculture** | ASC OA day 7 | ASC OA day 28 | BMSC OA day 7 | BMSC OA day 28 | Mixed OA day 7 | Mixed OA day 28 | Ch OA day 7 | Ch OA day 28 |
| **vs. ASC NB FB** | 0.3169 | 0.7484 | 0.3429 | 0.0317 * | 0.8857 | 0.3094 | 0.7302 | 0.0286 * |

**B) Quantification of collagens and proteoglycans in cell lysates (Fig. 3)**

| **Col I coculture** | BMSC OA day 28 | Mixed OA day 28 | Ch OA day 28 |
| --- | --- | --- | --- |
| **vs. monoculture** | 0.0025 ** | 0.0020 ** | 0.2118 |
| **Col II coculture** | BMSC OA day 28 | Mixed OA day 28 | Ch OA day 28 |
| **vs. monoculture** | 0.0052 ** | 0.5163 | < 0.0001 *** |
| **Col III coculture** | BMSC OA day 28 | Mixed OA day 28 | Ch OA day 28 |
| **vs. monoculture** | 0.0117 * | 0.0004 *** | 0.0293 * |
| **GAG coculture** | BMSC OA day 28 | Mixed OA day 28 | Ch OA day 28 |
| **vs. monoculture** | 0.0453 * | 0.0212 * | 0.0102 * |

**C) Quantification of cytokines, bFGF and GAGs in supernatants (Fig. 6)**

| **IL-1ß coculture** | ASC NB day 28 | ASC OA day 28 | BMSC OA day 28 | Mixed OA day 28 | Ch day OA 28 |
| --- | --- | --- | --- | --- | --- |
| **vs. monoculture** | 0.9273 | 0.0242 * | 0.0286 * | 0.0424 * | 0.0049 ** |
| **IL-6 coculture** | ASC NB day 7 | ASC OA day 7 | BMSC OA day7 | Mixed OA day 7 | Ch day OA 7 |
| **vs. monoculture** | 0.0007 *** | 0.1739 | 0.0014 ** | 0.0022 ** | o.ooo3 *** |
| **IL-8 coculture** | ASC NB day 7 | ASC OA day 7 | BMSC OA day7 | Mixed OA day 7 | Ch day OA 7 |
| **vs. monoculture** | < 0.0001 *** | 0.0948 | 0.0008 ** | 0.2240 | 0.0022 ** |
| **bFGF coculture** | ASC NB day 7 | ASC OA day 7 | BMSC OA day7 | Mixed OA day 7 | Ch day OA 7 |
| **vs. monoculture** | < 0.0001 *** | < 0.0001 *** | 0.0043 ** | 0.7483 | 0.0128 * |
| **GAG coculture** | ASC NB day 7 | ASC OA day 7 | BMSC OA day7 | Mixed OA day 7 | Ch day OA 7 |
| **vs. monoculture** | 0.0006 *** | < 0.0001 *** | 0.0021 ** | 0.0317 * | 0.0303 * |

| **IL-1ß coculture** | ASC OA day 28 | BMSC OA day 28 | Mixed OA day 28 | Ch OA day 28 |
| --- | --- | --- | --- | --- |
| **vs. ASC NB FB** | 0.3429 | 0.571 | 0.0061 ** | 0.0242 * |
| **IL-6 coculture** | ASC OA day 7 | BMSC OA day 7 | Mixed OA day 7 | Ch OA day7 |
| **vs. ASC NB FB** | 0.0020 ^##^ | 0.0031 ^##^ | 0.0005 ^###^ | < 0.0001 ^###^ |
| **IL-8 coculture** | ASC OA day 7 | BMSC OA day 7 | Mixed OA day 7 | Ch OA day7 |
| **vs. ASC NB FB** | 0.0002 ^###^ | 0.3154 | 0.1932 | 0.8749 |
| **bFGF coculture** | ASC OA day 7 | BMSC OA day 7 | Mixed OA day 7 | Ch OA day7 |
| **vs. ASC NB FB** | 0.3062 | 0.0005 ^###^ | 0.0559 | 0.7861 |
| **GAG coculture** | ASC OA day 7 | BMSC OA day 7 | Mixed OA day 7 | Ch OA day7 |
| **vs. ASC NB FB** | 0.8329 | 0.8708 | 0.8413 | 0.9307 |

**D) Quantification of cytokines, bFGF and GAGs supernatants of cell-free bone explants (Fig. 7)**

| **IL-1ß OA-** | subchondral bone day 7 | subchondral bone day 28 |
| --- | --- | --- |
| **vs. normal** | 0.0294 * | 0.2571 |
| **IL-6 OA-** | subchondral bone day 7 | subchondral bone day 28 |
| **vs. normal** | 0.1770 | 0.4693 |
| **IL-8 OA-** | subchondral bone day 7 | subchondral bone day 28 |
| **vs. normal** | 0.1770 | 0.1184 |
| **bFGF OA-** | subchondral bone day 7 | subchondral bone day 28 |
| **vs. normal** | 0.0017 ** | 0.0169 * |
| **GAG OA-** | subchondral bone day 7 | subchondral bone day 28 |
| **vs. normal** | 0.0173 * | 0.0037 ** |

| **IL-1ß day 7** | OA- subchondral bone | Normal subchondral bone |
| --- | --- | --- |
| **vs. day 28** | 0.3429 | 0.0422 * |
| **IL-6 day 7** | OA- subchondral bone | Normal subchondral bone |
| **vs. day 28** | 0.0024 ** | <0.0001 *** |
| **IL-8 day 7** | OA- subchondral bone | Normal subchondral bone |
| **vs. day 28** | 0.0024 ** | 0.0193 * |
| **bFGF day 7** | OA- subchondral bone | Normal subchondral bone |
| **vs. day 28** | 0.0260 * | 0.0014 ** |
| **GAG day 7** | OA- subchondral bone | Normal subchondral bone |
| **vs. day 28** | 0.8357 | 0.2222 |

**E) Stimulation of fibrin gel monocultures with IL-1ß, IL-6 or IL-8 (Fig. 8)**

| **ACAN + IL-1ß** | BMSC day 7 | Mixed day 7 | Ch day 7 |
| --- | --- | --- | --- |
| **unstimulated** | 0.0064 ** | 0.0095 ** | 0.3611 |
| **ACAN + IL-6** | BMSC day 7 | Mixed day 7 | Ch day 7 |
| **unstimulated** | 0.9337 | 0.9338 | 0.0055 ** |
| **ACAN + IL-8** | BMSC day 7 | Mixed day 7 | Ch day 7 |
| **unstimulated** | 0.0055 ** | 0.3611 | 0.3611 |

| **MMP2 + IL-1ß** | BMSC day 7 | Mixed day 7 | Ch day 7 |
| --- | --- | --- | --- |
| **unstimulated** | 0.0055 ** | 0.0054 ** | 0.3611 |
| **MMP2 +IL-6** | BMSC day 7 | Mixed day 7 | Ch day 7 |
| **unstimulated** | 0.3602 | 0.3611 | 0.9338 |
| **MMP2 + IL-8** | BMSC day 7 | Mixed day 7 | Ch day 7 |
| **unstimulated** | 0.3602 | 0.0037 ** | 0.9338 |

| **MMP3 + IL-1ß** | BMSC day 7 | Mixed day 7 | Ch day 7 |
| --- | --- | --- | --- |
| **unstimulated** | 0.037 ** | 0.0089 ** | 0.446 * |
| **MMP3 + IL-6** | BMSC day 7 | Mixed day 7 | Ch day 7 |
| **unstimulated** | 0.3173 | 0.0080 ** | 0.0054 ** |
| **MMP3 + IL-8** | BMSC day 7 | Mixed day 7 | Ch day 7 |
| **unstimulated** | 1.000 | 0.9338 | 0.3602 |

| **MMP13 + IL-1ß** | BMSC day 7 | Mixed day 7 | Ch day 7 |
| --- | --- | --- | --- |
| **unstimulated** | 0.0012 ** | 0.0002 *** | 0.0012 ** |
| **MMP13 +IL-6** | BMSC day 7 | Mixed day 7 | Ch day 7 |
| **unstimulated** | 0.0139 * | 0.9474 | 0.3871 |
| **MMP13 + IL-8** | BMSC day 7 | Mixed day 7 | Ch day 7 |
| **unstimulated** | 0.9334 | 0.0019 ** | 0.9561 |

**F) Quantification of gene expression in BMSC monocultures stimulated with OA bone explants conditioned medium (suppl. Fig. 3)**

| **Col I monoculture** | BMSC day 7 | BMSC day 28 |
| --- | --- | --- |
| **vs. conditioned medium** | 0.4762 | 0.0159 * |
| **Col II monoculture** | BMSC day 7 | BMSC day 28 |
| **vs. conditioned medium** | 1.0000 | 0.0286 * |
| **Col III monoculture** | BMSC day 7 | BMSC day 28 |
| **vs. conditioned medium** | 0.2571 | 0.0159 * |
| **Col X monoculture** | BMSC day 7 | BMSC day 28 |
| **vs. conditioned medium** | 0.2857 | 0.0159 * |

| **Col I day 7** | BMSC F | BMSC F +CM |
| --- | --- | --- |
| **vs. day 28** | 0.4103 | 0.8857 |
| **Col II day 7** | BMSC F | BMSC F+ CM |
| **vs. day 28** | 0.0095 ** | 0.0286 * |
| **Col III day 7** | BMSC F | BMSC F + CM |
| **vs. day 28** | 0.0381 * | 0.0159 * |
| **Col X day 7** | BMSC F | BMSC F + CM |
| **vs. day 28** | 0.0317 * | 0.3429 |

**Abbreviation list**

ACAN: aggrecan gene; ASC: adipose derived stem cells; bFGF: basic fibroblast growth factor; BMSC: bone marrow derived stem cells; ch: chondrocytes; CM: conditioned medium; Col I, II, III and X: Collagen I, II, III and X; F: monoculture (without subchondral bone explant); FB: co- and triculture with subchondral bone explant; GAG: glycosaminoglycan; IL-1ß: interleukin 1ß; IL-6: interleukin 6; IL-8: interleukin 8; Mixed: mixed cultures of BMSC and chondrocytes (1:1); MMP2: matrix metalloproteinase-2; MMP3: matrix metalloproteinase-3; MMP13: matrix metalloproteinase-13; NB: normal bone: OA: osteoarthritis; qPCR: quantitative polymerase chain reaction; vs.: versus

**Supplementary table 1: Statistical data (p-values)**

P-values of:

**(A) Cocultures vs. monocultures and normal vs. OA-subchondral bone cocultures.** *COL1A1*, *COL2A1*, *COL3A1* and *COL10A1* gene expression determined in ASC, BMSC, mixed cultures or chondrocytes kept as monocultures (F) or as co- and tricultures with osteoarthritic (OA FB) or normal subchondral bone explants (NB FB) at day 7 and day 28 (corresponding to Fig. 2).

**(B) Cocultures vs. monocultures.** Collagen protein (day 28) and proteoglycan (day 7) quantification in cell lysates from BMSC, mixed cultures or chondrocytes kept as monocultures (F) or as co- and tricultures with OA-subchondral bone explants (FB) (corresponding to Fig. 3).

**(C) Cocultures vs. monocultures and normal vs. OA-subchondral bone cocultures.** IL-1ß (day 28), IL-6 and -8, bFGF and soluble GAGs (all day 7) quantified in supernatants from ASC, BMSC, mixed cultures or chondrocytes kept as monocultures (F) or as co- and tricultures with osteoarthritic (OA FB) or normal subchondral bone explants (NB FB) (corresponding to Fig. 6).

**(D) Normal vs. OA- subchondral bone and day 7 vs. day 28.** IL-1ß, IL-6 and -8, bFGF and soluble GAGs quantified in supernatants from cell-free normal and OA-subchondral bone explants (corresponding to Fig. 7).

**(E) IL stimulated vs. unstimulated monocultures.** *ACAN, MMP2*, *MMP3* and *MMP13* gene expression at day 7 determined in BMSC, mixed cultures and chondrocytes stimulated for 7 days with 5ng/ml IL-1ß, 5ng/ml IL-6 or 10ng/ml IL-8 in chondrogenic medium (corresponding to Fig. 8).

**(F) Monocultures with unconditioned vs. conditioned medium and day 7 vs. day 28.** *COL1A1*, *COL2A1*, *COL3A1* and *COL10A1* gene expression in BMSC monocultures supplemented with OA- subchondral bone explant conditioned medium (corresponding to suppl. Fig. 3)

Stars (*) indicate significant differences between mono- and cocultures, hash signs (#) indicate significant differences between NB and OA cocultures. *p<0.05, **p<0.01, ***p<0.001; ^#^p<0.05, ^##^p<0.01, ^###^p<0.001
